# Depletion of acetate-producing bacteria from the gut microbiota facilitates cognitive impairment through the gut-brain neural mechanism in diabetic mice

**DOI:** 10.1186/s40168-021-01088-9

**Published:** 2021-06-25

**Authors:** Hong Zheng, Pengtao Xu, Qiaoying Jiang, Qingqing Xu, Yafei Zheng, Junjie Yan, Hui Ji, Jie Ning, Xi Zhang, Chen Li, Limin Zhang, Yuping Li, Xiaokui Li, Weihong Song, Hongchang Gao

**Affiliations:** 1 Institute of Metabonomics & Medical NMR, School of Pharmaceutical Sciences, Wenzhou Medical University, Wenzhou, 325035 China; 2 Department of Pulmonary and Critical Care Medicine, The First Affiliated Hospital of Wenzhou Medical University, Wenzhou, 325015 China; 3 Institute of Aging, School of Mental Health, Wenzhou Medical University, Wenzhou, 325035 China; 4 State Key Laboratory of Magnetic Resonance and Atomic and Molecular Physics, Wuhan Institute of Physics and Mathematics, Chinese Academy of Sciences, Wuhan, 430070 China

**Keywords:** Acetate, Metabolome, Diabetes, Cognition, Microbiome, Gut-brain axis

## Abstract

**Background:**

Modification of the gut microbiota has been reported to reduce the incidence of type 1 diabetes mellitus (T1D). We hypothesized that the gut microbiota shifts might also have an effect on cognitive functions in T1D. Herein we used a non-absorbable antibiotic vancomycin to modify the gut microbiota in streptozotocin (STZ)-induced T1D mice and studied the impact of microbial changes on cognitive performances in T1D mice and its potential gut-brain neural mechanism.

**Results:**

We found that vancomycin exposure disrupted the gut microbiome, altered host metabolic phenotypes, and facilitated cognitive impairment in T1D mice. Long-term acetate deficiency due to depletion of acetate-producing bacteria resulted in the reduction of synaptophysin (SYP) in the hippocampus as well as learning and memory impairments. Exogenous acetate supplement or fecal microbiota transplant recovered hippocampal SYP level in vancomycin-treated T1D mice, and this effect was attenuated by vagal inhibition or vagotomy.

**Conclusions:**

Our results demonstrate the protective role of microbiota metabolite acetate in cognitive functions and suggest long-term acetate deficiency as a risk factor of cognitive decline.

**Video Abstract**

**Supplementary Information:**

The online version contains supplementary material available at 10.1186/s40168-021-01088-9.

## Background

Type 1 diabetes mellitus (T1D) is an autoimmune disease characterized by hyperglycemia due to pancreatic β-cell destruction and insulin secretion impairment [1]. Data from the International Diabetes Federation (IDF) showed that over one million children and adolescents suffered from T1D all over the world in 2017 [2]. T1D causes a series of diabetic complications [3], of which cognitive dysfunction receives less attention but seriously affects the quality of life of patients [4]. Moreover, T1D patients had a significantly higher risk of dementia than T2D patients [5]. Therefore, diabetes specialists have recommended guidelines on the screening and management of T1D-related cognitive injury in daily practice [4].

In recent years, diabetes-related cognitive dysfunction has gotten major attention [6], but the potential mechanisms underlying T1D-related cognitive decline remain elusive, severely affecting its diagnosis and treatment in clinical practice. Several studies suggested that cognitive disorders in patients with T1D might be attributed to changes in cerebral structures, such as brain atrophy [7], reduced white matter volume [8], and lower gray matter density [9]. Disrupted brain functional connectivity may adversely affect cognitive performances in T1D patients [10, 11]. In animal studies, T1D-related cognitive decline has been linked to neuronal apoptosis [12, 13], oxidative stress [14], inflammation [15], and metabolic disorders [16, 17].

Gut microbiota has been associated with the development of T1D [18, 19]. We previously reported that the gut microbiota was disrupted in T1D rats with cognitive decline [20]. Modification of the gut flora by vancomycin [21] or methoxyl pectin [22] decreased T1D incidence in non-obese diabetic mice. In the current study, we used a non-absorbable antibiotic vancomycin to modify the gut microbiota in streptozotocin (STZ)-induced T1D mouse model and examined the effect of microbial shifts on cognitive functions in T1D mice. However, we found that vancomycin-induced reductions in acetate-producing bacteria accelerated cognitive decline in T1D mice. Long-term acetate deficiency reduced hippocampal synaptophysin and facilitated cognitive impairments in mice. Our results reveal the important role of microbial metabolite acetate in cognitive functions by regulating the gut-brain axis.

## Results

### Vancomycin exposure accelerates cognitive dysfunction in T1D mice

Streptozotocin (STZ) as a toxic glucose analogue can enter pancreatic β-cell via the glucose transporter GLUT2, cause β-cell necrosis and in turn develop into type 1 diabetes mellitus (T1D) [23]. In the current study, C57BL/6 mice were treated with multiple-low-dose STZ to develop the animal model of T1D. Expectedly, STZ-treated mice exhibited typical of T1D phenotypes including significantly higher blood glucose level (Figure S1a), daily food intake (Figure S1b), and daily water intake (Figure S1c) as well as significantly lower body weight (Figure S1d) relative to normal control (CON) mice. Moreover, STZ treatment significantly decreased the number of insulin-producing cells but increased glucagon-producing cells (Figure S1e). The concentration of serum insulin was also significantly reduced in STZ-treated mice (Figure S1f, *P*<0.001).

**Fig. 1:**
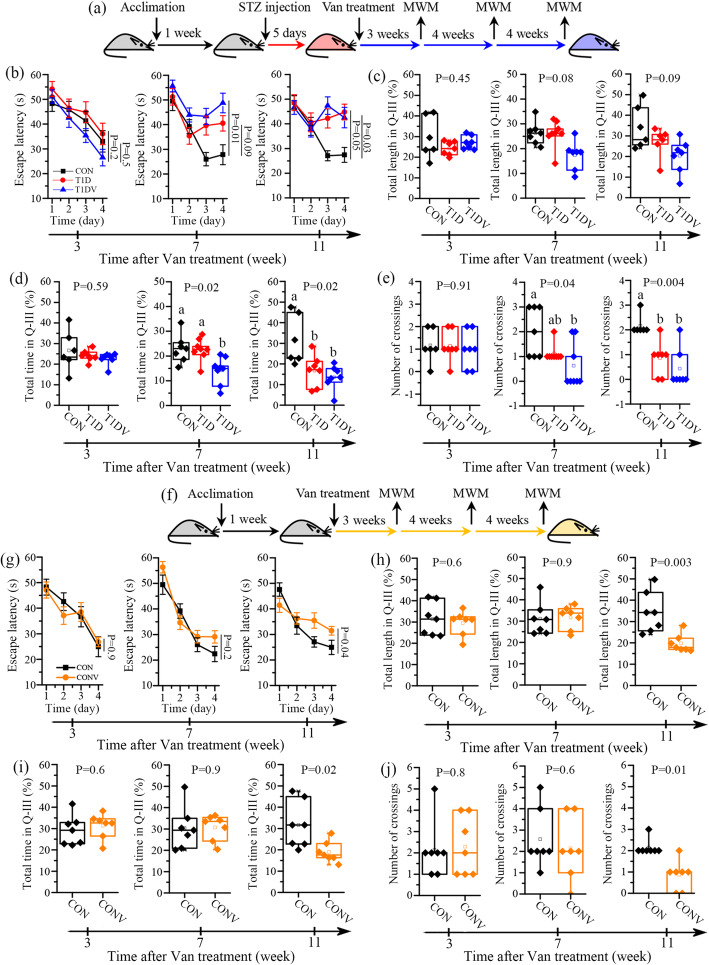
Vancomycin exposure accelerates learning and memory impairment in mice. **a** After a 1-week acclimation, mice were injected with streptozocin for 5 consecutive days to develop type 1 diabetic (T1D) mice and then administered with vancomycin (Van). The Morris water maze (MWM) test was used to evaluate learning and memory ability in T1D mice at 3, 7, and 11 weeks after Van treatment (*n*=6–7 mice per group). **b** Escape latency during the training period in normal control (CON), T1D, and Van-treated T1D (T1DV) mice at 3, 7, and 11 weeks. **c** Percentage of total swimming length in the Q-III area (original platform quadrant), **d** percentage of total swimming time in the Q-III area, and **e** the number of crossings over the original platform location during the test period in CON, T1D, and T1DV mice at 3, 7, and 11 weeks. **f** After a 1-week acclimation, mice were treated with Van and then their learning and memory ability was assessed by the MWM test at 3, 7, and 11 weeks after Van treatment (*n*=6–7 mice per group). **g** Escape latency during the training period in normal control (CON) and Van-treated CON (CONV) mice at 3, 7, and 11 weeks. **h** Percentage of total swimming length in the Q-III area, **i** percentage of total swimming time in the Q-III area, and **j** the number of crossings over the original platform location during the test period in CON and CONV mice at 3, 7, and 11 weeks. Time-series data were analyzed by repeated measures one-way ANOVA followed by Bonferroni’s multiple comparisons test. The differences among three groups were analyzed by one-way ANOVA with Bonferroni’s multiple comparisons test, and data with different lowercase codes are significantly different (P < 0.05). The difference between two groups was determined by two-tailed unpaired Student’s t test with Bonferroni correction

To determine whether the gut microbiota modification affects cognitive functions, T1D mice were orally administered with non-absorbable broad-spectrum antibiotic vancomycin. Vancomycin exposure did not alter diabetes characteristics in T1D mice, excepting a decrease in daily water intake probably due to its bitter taste (Figure S1c). The Morris water maze (MWM) test was utilized to assess learning and memory ability in mice at 3, 7, and 11 weeks (Fig. 1a). At 3 weeks, there was no statistically significant difference in the escape latency during the training phase among CON, T1D, and vancomycin-treated T1D (T1DV) mice (Fig. 1b). However, T1DV mice (P=0.01), but not T1D mice (P=0.09), had longer escape latency than CON mice at 7 weeks, indicating an impaired learning ability after vancomycin treatment. At 11 weeks, relative to CON mice, the escape latency was significantly longer in both T1D (P=0.05) and T1DV (P=0.03) mice (Fig. 1b). After 4 days of training, the escape platform was removed to evaluate the memory performance of mice. The swimming trajectories in CON, T1D, and T1DV mice during the test phase are illustrated in Figure S2. We observed that the percentages of the total swimming length (Fig. 1c, P=0.08) and time (Fig. 1d, P=0.02) in the original platform area and the number of crossings over the original platform location (Fig. 1e, P=0.04) were significantly decreased in T1DV mice at 7 weeks, but not in T1D mice, when compared with CON mice. There were no significant differences in these behavioral parameters at 3 weeks among these three groups (Fig. 1c–e). In addition, cognitive injury was also observed in T1D mice at 11 weeks, suggesting that long-term high blood glucose resulted in diabetic cerebral complication. However, it is worth noting that T1DV mice showed a slight but not significant reduction in memory ability relative to T1D mice at 11 weeks (Fig. 1c–e). Collectively, vancomycin exposure facilitated cognitive impairment in T1D mouse model. To examine the influence of vancomycin exposure on inflammatory response, TNF-α and IL-10 were detected in the hippocampus at 7 weeks by using immunohistochemistry (Figure S3). The results demonstrate that there were no significant differences in TNF-α and IL-10 in the hippocampus including CA1, CA3, and DG regions among CON, T1D, and T1DV mice, suggesting that vancomycin-induced cognitive decline was not attributed to hippocampal inflammation in T1D mice at 7 weeks.

Subsequently, we treated normal healthy mice with vancomycin (CONV) to modify the gut microbiota and then evaluated their cognitive functions at 3, 7, and 11 weeks after vancomycin treatment (Fig. 1f). The results show that CONV mice had significantly longer escape latency than CON mice at 11 weeks (P=0.04) but not at 3 and 7 weeks (P>0.05) during the training period (Fig. 1g). Figure S4 illustrates the swimming trajectories in CON and CONV mice during the test period. We found that the percentages of the total swimming length (Fig. 1h, P=0.003) and time (Fig. 1i, P=0.02) in the original platform area and the number of crossings over the original platform location (Fig. 1j, P=0.01) were significantly reduced in CONV mice relative to CON mice at 11 weeks but not at 3 and 7 weeks after vancomycin exposure (Fig. 1h–j). Our results imply that long-term exposure to vancomycin also impaired cognitive performances in normal healthy mice. According to the behavioral results of CON and T1D mice after vancomycin treatment, moreover, we speculate that multiple perturbations of the gut microbiome can potentially accelerate and aggravate the impairment of learning and memory in mice. Yet, such a hypothesis still needs to be further investigated and verified.

### Vancomycin decreases acetate-producing bacteria and fatty acid biosynthesis in T1D mice

We then investigated vancomycin effect on the structure and function of the gut microbiota using 16S rRNA and metagenomic sequencing analyses. In microbiome analysis, an average number of 90,226 total reads of microbial 16S rDNA were acquired from fecal samples by using Illumina sequencing, of which the combined reads were 87,511 and the combined percentage was 97.0%. In metagenomic sequencing, a total of 98,312.02 Mbp of raw data (average, 8192.67 Mbp) were acquired from fecal samples. After data pretreatment, 97,889.42 Mbp (average, 8157.45 Mpb) of total clean data and 99.57% of the effective rate were obtained in this study.

**Fig. 2:**
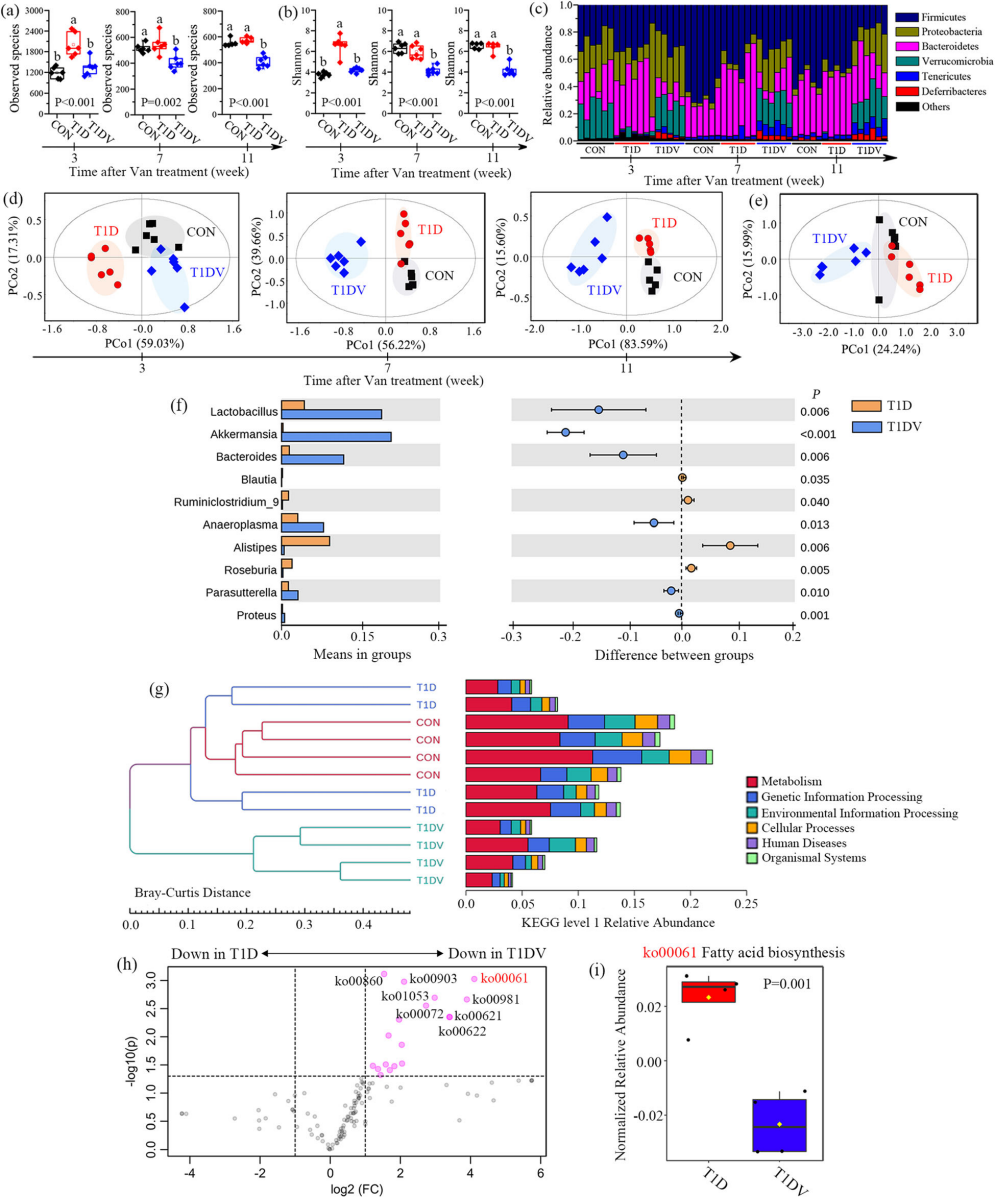
Vancomycin exposure alters the structure and function of the gut microbiota in mice. **a** The observed species and **b** Shannon index in normal control (CON), type 1 diabetic (T1D), and vancomycin-treated T1D (T1DV) mice at 3, 7, and 11 weeks (*n*=5–6 mice per group). **c** Relative abundance of the gut microbiota at the phylum level in cecum contents of CON, T1D, and T1DV mice at 3, 7, and 11 weeks (*n*=5–6 mice per group). **d** PCoA-based classification using the gut microbiome at the phylum level in cecum contents of CON, T1D, and T1DV mice at 3, 7, and 11 weeks (*n*=5–6 mice per group). **e** PCoA-based classification using the gut microbiome at the genus level in cecum contents of CON, T1D, and T1DV mice at 7 weeks (*n*=5–6 mice per group). **f** Top 10 microbes that significantly altered between T1D and T1DV mice at 7 weeks. **g** Cluster analysis based on Bray-Curtis distance using the predicted KEGG orthology abundances at level 1 of the gut microbiota in cecum contents of CON, T1D, and T1DV mice at 7 weeks. **h** Volcano plot analysis based on metabolism-related functions of the gut microbiota in cecum contents between T1D and T1DV mice at 7 weeks. **i** The change of fatty acid biosynthesis on the gut microbiota in cecum contents between T1D and T1DV mice at 7 weeks (*n*=4 mice per group). The difference between two groups was determined by two-tailed unpaired Student’s t test with Bonferroni correction. The differences among three groups were analyzed by one-way ANOVA with Bonferroni’s multiple comparisons test, and data with different lowercase codes are significantly different (P < 0.05)

The results reveal that the observed species (Fig. 2a) and diversity (Fig. 2b, Shannon index) of the gut microbiota in cecum contents was significantly increased in T1D mice relative to CON and T1DV mice at 3 weeks after vancomycin treatment. However, vancomycin exposure for 7 and 11 weeks resulted in significant reductions in the observed species and diversity of the gut microbiota in T1DV mice when compared with CON and T1D mice (Fig. 2a, b). Figure 2c illustrates an overview of microbial profiling at the phylum level, where we found that the microbial composition of T1D mice was differed from CON and T1DV mice at 3 weeks. Yet, a distinct difference in the microbial composition was observed in T1DV mice relative to CON and T1D mice at both 7 and 11 weeks after vancomycin treatment, as indicated by increases in the relative abundances of *Proteobacteria* and *Verrucomicrobia* but decreases in the relative abundances of *Firmicutes* and *Bacteroidetes* (Fig. 2c). Subsequently, PCoA was used to evaluate the discrimination between different groups based on the gut microbiome (Fig. 2d). We found that vancomycin treatment (T1DV) was clearly separated from the other two groups especially at 7 and 11 weeks (Fig. 2d). Moreover, it can be seen from PCoA results at the genus level that apparent separations were obtained among CON, T1D, and T1DV mice at 3 (Figure S5a), 7 (Fig. 2e), and 11 (Figure S5b) weeks. The results demonstrate that vancomycin-driven shifts in the gut microbiota of T1D mice included higher relative abundances of *Akkermansia*, *Lactobacillus*, and *Parasutterella* at 3 (Figure S5c), 7 (Fig. 2f), and 11 (Figure S5d) weeks. Vancomycin exposure also significantly reduced acetate-producing bacteria including *Alistipes*, *Blautia*, *Ruminclostridium_9*, and *Roseburia* in T1D mice at all three time points (Fig. 2f; Figures S5c and S5d). Besides, PCoA results show that the gut microbial patterns in CON mice were significantly altered after vancomycin treatment at both the phylum (Figure S6a) and genus (Figure S6b) levels. Acetate-producing bacteria, such as *Alistipes*, *Blautia*, *Ruminclostridium_9*, and *Roseburia*, were also identified to be significantly decreased in cecum contents of CON mice treated with vancomycin (Figure S7).

To further investigate the impact of vancomycin on the gut microbiota functions, we performed a metagenomic analysis of the bacteria in cecum contents and annotated the metagenomic reads based on the KEGG database. Figure 2g illustrates that T1DV mice were clearly differed from CON and T1D mice, suggesting disrupted gut microbiota functions after vancomycin exposure. Furthermore, we carried out a volcano plot analysis based on the metabolic functions of the gut flora, as shown in Fig. 2h. The results reveal that vancomycin significantly decreased the metabolic ability of the gut microbiota in T1D mice (Fig. 2h), of which fatty acid biosynthesis was identified as one of mainly affected microbial metabolisms (Fig. 2i).

### Long-term acetate deficiency causes cognitive decline in mice

To examine vancomycin effect on host metabolism, the metabolic profiles of feces, cecum contents, cecum and colonic tissues, serum, and the hippocampus in mice were analyzed by using a ^1^H NMR-based metabolomics method. Typical NMR spectra are illustrated in Figure S8, where we identified 41 metabolites involving amino acids, fatty acids, organic acids, nucleosides, and others.

**Fig. 3:**
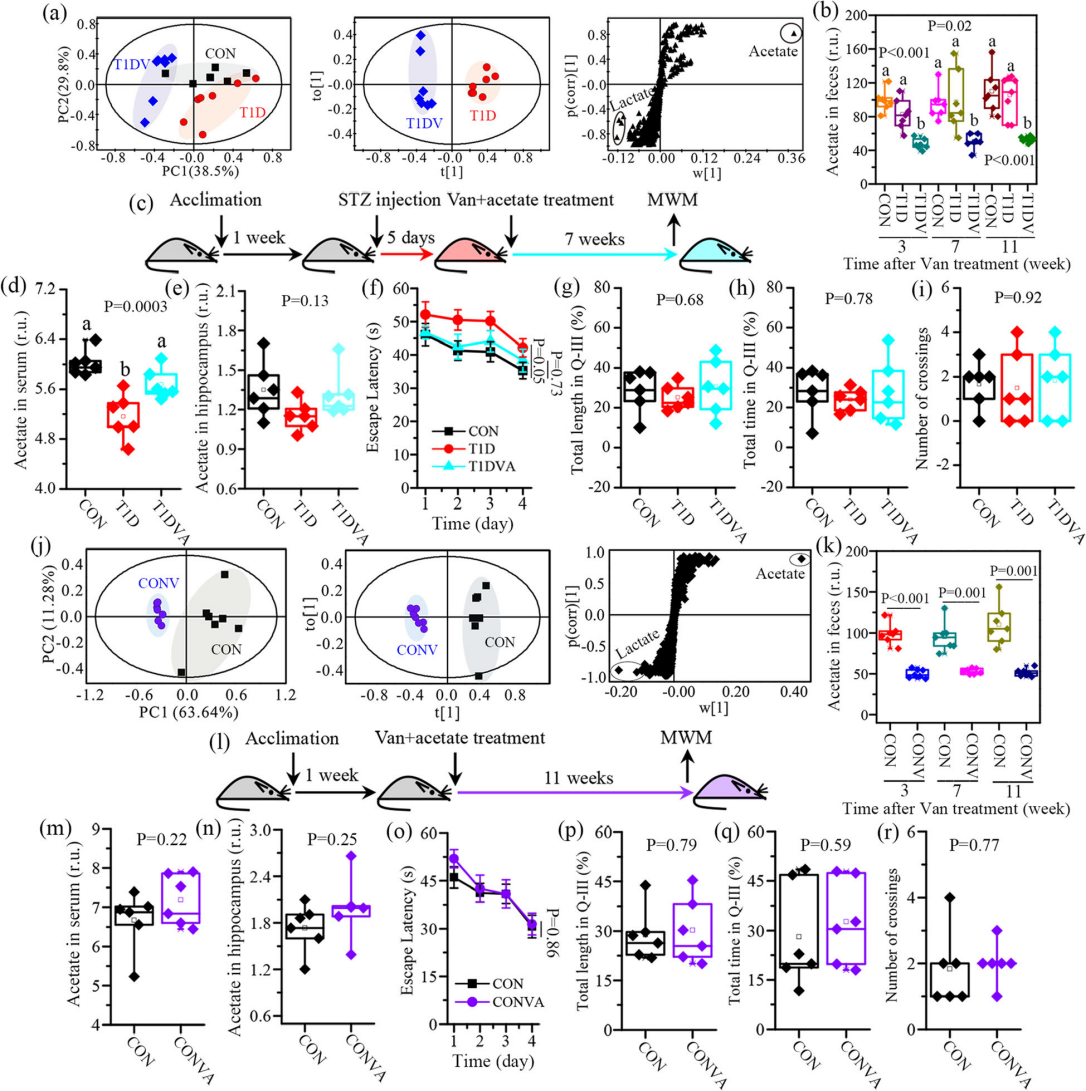
Exogenous acetate supplement improves cognitive ability in mice with gut microbiota dysbiosis induced by vancomycin. **a** PCA-based classification using fecal metabolomic profiling in normal control (CON), type 1 diabetic (T1D), and vancomycin-treated T1D (T1DV) mice at 7 weeks. OPLS-DA-based classification and its corresponding S-plot using fecal metabolomic profiling between T1D and T1DV at 7 weeks (*n*=6 mice per group). **b** The change of acetate level in feces of CON, T1D, and T1DV mice at 3, 7, and 11 weeks (*n*=6 mice per group). **c** After a 1-week acclimation, mice were injected with streptozocin for 5 consecutive days to develop T1D mice and then treated with vancomycin (Van) plus acetate. The Morris water maze (MWM) test was used to evaluate learning and memory ability in T1D mice at 7 weeks after Van treatment (*n*=5–6 mice per group). **d**, **e** The change in acetate level in serum and the hippocampus of CON, T1D, and T1DV treated with acetate (T1DVA) mice for 7 weeks. **f** Escape latency during the training period in CON, T1D, and T1DVA mice at 7 weeks. **g** Percentage of total swimming length in the Q-III area (original platform quadrant), **h** percentage of total swimming time in the Q-III area, and **i** the number of crossings over the original platform location during the test period in CON, T1D, and T1DVA mice at 7 weeks. **j** PCA-based classification using fecal metabolomic profiling between normal control (CON) and vancomycin-treated CON (CONV) mice at 11 weeks. OPLS-DA-based classification and its corresponding S-plot using fecal metabolomic profiling between T1D and CONV at 11 weeks. **k** The change of acetate level in feces of CON and CONV mice at 3, 7, and 11 weeks. **l** After a 1-week acclimation, mice were treated with Van plus acetate and then their learning and memory ability was assessed by the MWM test at 11 weeks after Van treatment. **m**, **n** The change in acetate level in serum and the hippocampus of CON and CONV treated with acetate (CONVA) mice for 11 weeks. **o** Escape latency during the training period in CON and CONVA mice at 11 weeks. **p** Percentage of total swimming length in the Q-III area (original platform quadrant), **q** percentage of total swimming time in the Q-III area, and **r** the number of crossings over the original platform location during the test period in CON and CONVA mice at 11 weeks. Time-series data were analyzed by repeated measures one-way ANOVA followed by Bonferroni’s multiple comparisons test. The difference between two groups was determined by two-tailed unpaired Student’s t test with Bonferroni correction. The differences among three groups were analyzed by one-way ANOVA with Bonferroni’s multiple comparisons test, and data with different lowercase codes are significantly different (P < 0.05). The difference between two groups was determined by two-tailed unpaired Student’s t test with Bonferroni correction

Principal component analysis (PCA) was used to examine the distinctions of metabolic patterns between different groups based on ^1^H NMR-derived metabolomics data. The results demonstrate that the fecal metabolic pattern in T1DV mice was clearly separated from the other two groups at 7 weeks (Fig. 3a). Additionally, differences in the metabolic patterns were also observed in feces (Figure S9a), cecum contents (Figure S9b), cecum tissues (Figure S9c), colonic tissues (Figure S9d), and serum (Figure S9e) of T1DV mice relative to CON and T1D mice at all three time points, suggesting that vancomycin exposure altered metabolic phenotypes in T1D mice. However, these metabolic differences were not noticeable in the hippocampus (Figure S9f). Afterward, orthogonal partial-least-squares discriminant analysis (OPLS-DA) was employed to identify metabolic changes between T1D and T1DV mice at 7 weeks, since at this time point vancomycin treatment accelerated cognitive decline in T1D mice. We found that clear and robust separations were obtained between these two groups using the metabolomes of feces (Fig. 3a) and other samples studied herein (Figure S10). The results from the corresponding S-plots show that acetate is a shared metabolite that mainly contributed to the separations of metabolic phenotypes between T1D and T1DV mice (Fig. 3a; Figure S10). We then quantified the relative concentration of acetate in feces (Fig. 3b), cecum contents (Figure S11a), cecum tissues (Figure S11b), colonic tissues (Figure S11c), serum (Figure S11d), and the hippocampus (Figure S11e). The results reveal that vancomycin treatment significantly reduced acetate level and the descent rate ranged from 20.4 to 57.5% at 7 weeks, which might be attributed to vancomycin-induced reductions in acetate-producing bacteria shown in Fig. 2f. Moreover, there were no significant differences in acetate level between CON and T1D mice at 7 weeks (Fig. 3b). In addition, the levels of butyrate and propionate, another two major SCFAs, were also significantly decreased in feces (Figures S12a and S12e), cecum contents (Figures S12b and S12f), and cecum tissues (Figures S12c and S12g) of T1D mice at 3, 7, and 11 weeks after vancomycin treatment, although their alterations were not obvious in colonic tissues (Figures S12d and S12h).

To verify whether decreased acetate level affects cognitive functions, T1DV mice were fed with acetate in their drinking water for 7 weeks (Fig. 3c). Administration of acetate increased vancomycin-induced reduction in acetate level in serum (Fig. 3d, P=0.0003) and the hippocampus (Fig. 3e, P=0.13). The MWM test show that the escape latency of T1D mice was longer than that of CON mice (P=0.05) but acetate-fed T1DV (T1DVA) mice were getting close to CON mice, as illustrated in Fig. 3f. In addition, their swimming trajectories during the test period are illustrated in Figure S13a, where we found that T1DV mice did not display statistically significant decreases in the percentage of the total swimming length (Fig. 3g, P=0.68) and time (Fig. 3h, P=0.78) in the original platform area as well as the number of crossings over the original platform location (Fig. 3i, P=0.92) relative to T1D mice at 7 weeks after exogenous acetate supplement.

To investigate the impact of the other two short-chain fatty acids (SCFAs) on cognitive functions, T1DV mice were fed with butyrate (T1DVB) or propionate (T1DVP) in their drinking water for 7 weeks. The results demonstrate that the escape latency was not significantly altered among CON, T1D, T1DVB, and T1DVP mice (Figure S14a). Moreover, according to their swimming trajectories during the test phase (Figure S14b), T1DV mice after exogenous butyrate administration did not have significant decreases in the percentage of the total swimming length (Figure S14c, *P*=0.31) and time (Figure S14d, *P*=0.29) in the original platform area when compared with T1D mice, which is similar with the effect of acetate (Fig. 3g, h). However, exogenous propionate treatment showed a decrease trend in these two behavioral parameters relative to other groups. Moreover, the number of crossings over the original platform location was still relatively lower in T1DVB and T1DVP mice than that in T1D mice and significantly decreased relative to CON mice (Figure S14e, P=0.04). Taken together, these findings indicate that SCFAs could alleviate vancomycin-promoted cognitive decline in T1D mice, but acetate may possess a relatively better effect.

Furthermore, we found that vancomycin exposure significantly altered the faecal metabolic phenotypes in CON mice at 3 (Figure S15a), 7 (Figure S15b), and 11 (Fig. 3j) weeks based on PCA and OPLS-DA models, and acetate was identified as an important metabolite from S-plots. The relative level of acetate in feces was significantly reduced in CON mice after vancomycin treatment (Fig. 3k) probably due to decreases in acetate-producing bacteria. To study whether acetate treatment alleviates vancomycin-induced cognitive decline in normal healthy mice, CON mice were fed with vancomycin plus administration of acetate (CONVA) in their drinking water for 11 weeks (Fig. 3l). There was no significant difference in acetate level in serum (Fig. 3m, P=0.22) and the hippocampus (Fig. 3n, P=0.25) between CON and CONVA mice, suggesting that exogenous acetate supplement effectively recovered acetate level in mice treated with vancomycin. The MWM test reveals that CONVA mice had a similar trend in the escape latency during the training period compared with CON mice (Fig. 3o). Their swimming trajectories during the test period are showed in Figure S13b, where no significant differences in the percentage of the total swimming length (Fig. 3p, P=0.79) and time (Fig. 3q, P=0.59) in the original platform area and the number of crossings over the original platform location (Fig. 3r, P=0.77) between CON and CONVA mice. These findings suggest that acetate plays an important role in vancomycin-induced cognitive decline in mice.

### Exogenous acetate restores vancomycin-induced reduction of hippocampal SYP level in T1D mice

**Fig. 4:**
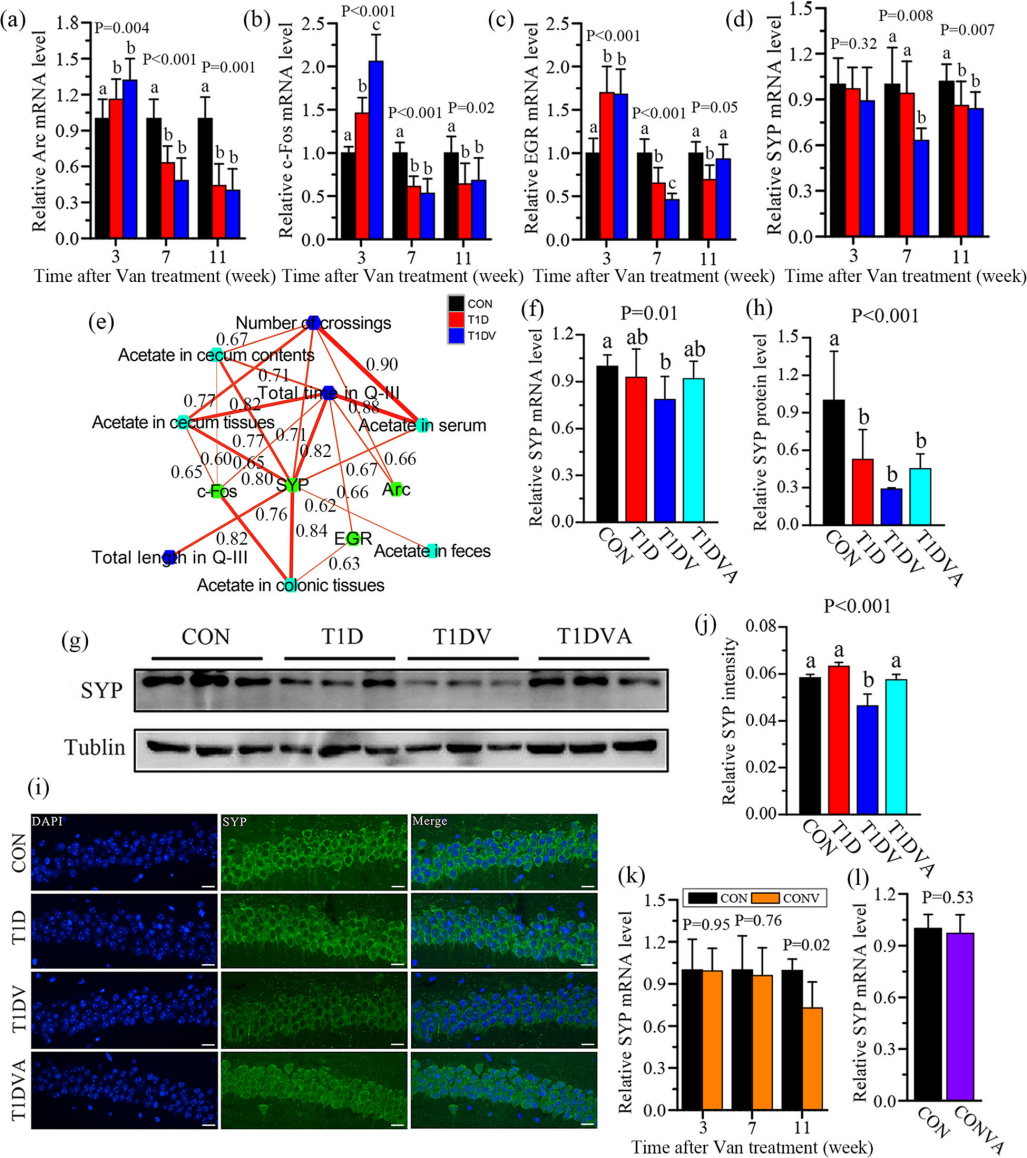
Exogenous acetate supplement increases hippocampal synaptophysin level in mice with gut microbiota dysbiosis induced by vancomycin. The changes in the mRNA expression levels of **a** Arc, **b** c-Fos, **c** EGR, and **d** SYP in normal control (CON), type 1 diabetic (T1D), and vancomycin-treated T1D (T1DV) mice at 3, 7, and 11 weeks. **e** Correlation network analysis among acetate, cognitive parameters, and synaptic plasticity-related genes. The relationship was analyzed by Pearson’s correlation and red line represents positive correlation. **f** Relative SYP mRNA expression level in the hippocampus of CON, T1D, T1DV, and T1DV treated with acetate (T1DVA) mice at 7 weeks. **g**, **h** The level of SYP protein detected by Western blotting and the corresponding quantitative data in the hippocampus of CON, T1D, T1DV, and T1DVA mice at 7 weeks (*n*=3 mice per group). **i**, **j** Representative SYP immunostaining and the quantified relative SYP intensity in hippocampal CA1 region of CON, T1D, T1DV, and T1DVA mice at 7 weeks (*n*=3 mice per group). Scale bar = 400 μm. **k**, **l** The change in the relative SYP mRNA expression level between CON and vancomycin-treated CON (CONV) mice at 3, 7, and 11 weeks and between CON and CONV treated with acetate (CONVA) mice at 11 weeks. Data are presented as mean±s.d. of *n*=5–6 mice per group. The difference between two groups was determined by two-tailed unpaired Student’s t test with Bonferroni correction. The differences among three or four groups were analyzed by one-way ANOVA with Bonferroni’s multiple comparisons test, and data with different lowercase codes are significantly different (P < 0.05)

Synaptic plasticity has been regarded as the basis for learning and memory [24]. We next examined the expression of several synaptic plasticity-related genes (e.g., Arc, c-Fos, EGR, and SYP) in the hippocampus of mice using qRT-PCR method. At 7 weeks, the mRNA expression levels of Arc (Fig. 4a), c-Fos (Fig. 4b), and EGR (Fig. 4c) in the hippocampus were significantly decreased in T1D and T1DV mice compared with CON mice (P<0.001). However, T1DV mice had a significant lower mRNA expression level of hippocampal SYP than the other two groups and no significant difference was detected between CON and T1D mice at 7 weeks (Fig. 4d). At 11 weeks, relative to CON mice, the mRNA expression level of SYP was significantly reduced in the hippocampus of T1D and T1DV mice (Fig. 4d, P=0.007). Correlation network analysis illustrates that hippocampal SYP gene had much more positive correlations with acetate and cognitive parameters than other synaptic plasticity-related genes (Fig. 4e, R=0.62-0.84).

SYP is a synaptic vesicle membrane protein that accounts for approximately 7–10% of the total vesicle protein [25] and used as a marker for synaptogenesis and synaptic density [26]. SYP-knockout mice showed a significant dysfunction in learning and memory relative to wild-type mice [27], confirming the role of SYP in modulating cognitive functions. To determine whether acetate affects hippocampal SYP level, T1DV mice were then fed with acetate in their drinking water for 7 weeks (Fig. 3c). The results reveal that exogenous acetate supplement increased the mRNA and protein levels of SYP in the hippocampus of T1DV mice (Fig. 4f–h). Using immunofluorescence analysis, we also found that acetate treatment for 7 weeks significantly increased the SYP level in hippocampal CA1 area of T1DV mice (Fig. 4i, j; P<0.001). Yet, exogenous butyrate or propionate consumption for 7 weeks did not increase hippocampal SYP level at both mRNA and protein levels (Figure S16). In CON mice, a significant reduction in hippocampal SYP level was detected at 11 weeks after vancomycin treatment but not at 3 and 7 weeks (Fig. 4k), consistent with the MWM results that impaired learning and memory occurred in normal healthy mice until week 11 of vancomycin exposure (Fig. 1g–j). The level of SYP in the hippocampus was recovered to the normal level in CONV mice with exogenous acetate supplement (Fig. 4l).

### Fecal microbiota transplant recovered acetate and hippocampal SYP in T1DV mice

**Fig. 5:**
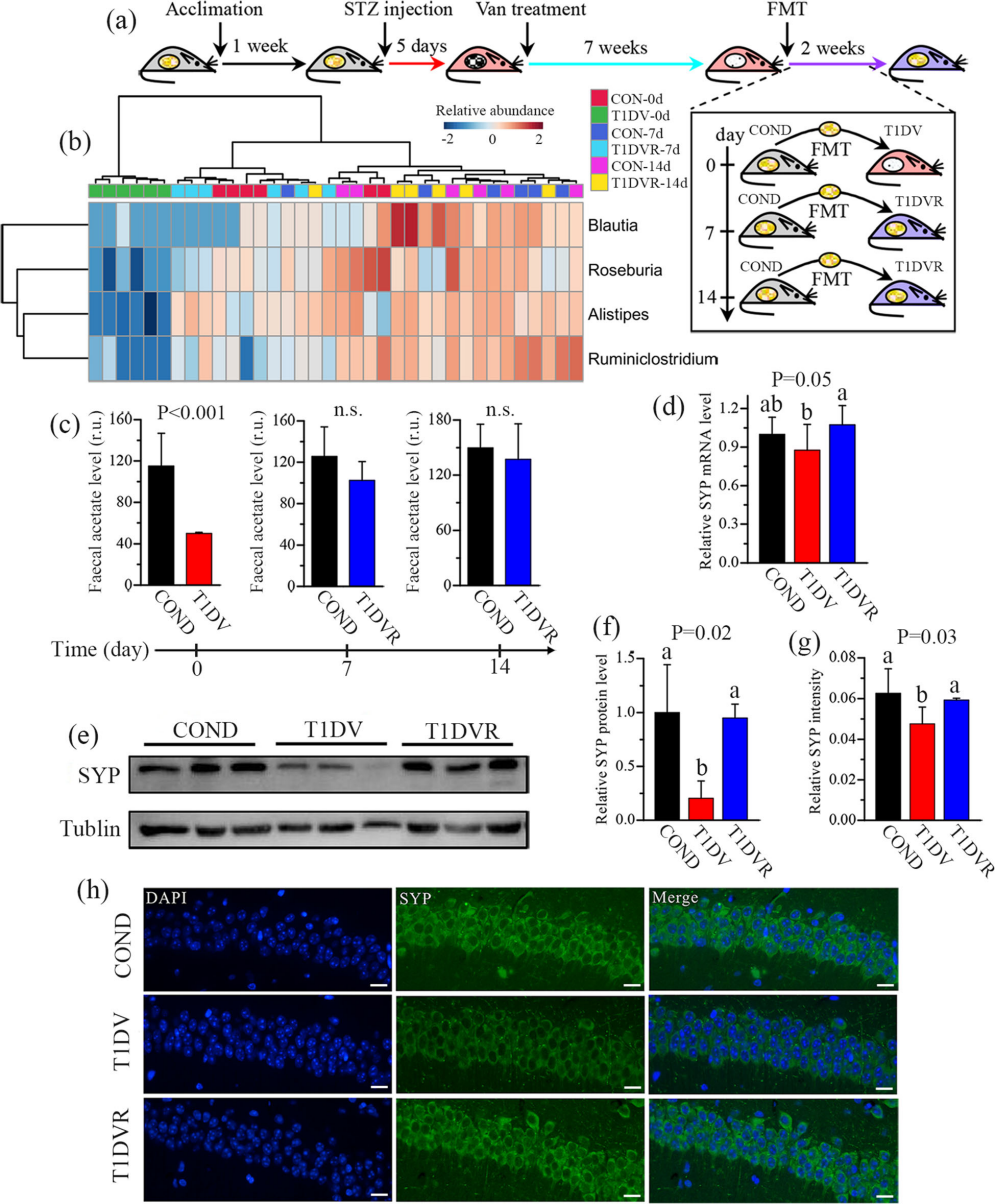
Fecal microbiota transplant improves hippocampal synaptophysin level in vancomycin-treated type 1 diabetic mice. **a** Schematic drawing of fecal microbiota transplant (FMT). After a 1-week acclimation, mice were injected with streptozocin for 5 consecutive days to develop type 1 diabetic (T1D) mice and then administered with vancomycin (Van) for 7 weeks. Subsequently, fecal material from healthy age-matched donor mice (COND) was transferred to Van-treated T1D (T1DV) recipients (T1DVR) for 14 consecutive days. **b** The changes in acetate-producing bacteria in T1DV mice during FMT. **c** The change of fecal acetate level in T1DV mice during FMT. **d** Relative SYP mRNA expression level in the hippocampus of COND, T1DV, and T1DVR mice. **e**, **f** Relative SYP protein level detected by Western blotting and the corresponding quantitative data in the hippocampus of COND, T1DV, and T1DVR mice (*n*=3 mice per group). **g**, **h** Representative SYP immunostaining and the quantified relative SYP intensity in hippocampal CA1 region of CON, T1D, T1DV, and T1DVA mice at 7 weeks. Scale bar = 400 μm. Data are presented as mean±s.d. of *n*=6 mice per group. The difference between two groups was determined by two-tailed unpaired Student’s t test with Bonferroni correction. The differences among three groups were analyzed by one-way ANOVA with Bonferroni’s multiple comparisons test, and data with different lowercase codes are significantly different (P < 0.05). n.s., no significant difference

To further verify the role of the gut microbiota in hippocampal synaptic plasticity, fecal microbiota from healthy donor mice (COND) were transferred to T1DV recipients for 14 days (Fig. 5a). The results show that the observed species (Figure S17a) and Shannon index (Figure S17b) of the gut microbiota in feces was significantly lower in T1DV mice relative to COND mice. However, these differences were gradually diminished after fecal microbiota transplant (FMT), especially for Shannon index (Figure S17b). It can be seen from PCoA results that the gut microbial pattern at the phylum level in feces of T1DV mice was clearly separated from that of COND mice (Figure S17c). Of note, T1DV mice after FMT (T1DVR) were getting close to COND mice (Figures S17d and S17e), indicating that the gut microbiota in T1DV mice was partly reshaped by FMT from COND mice. Compared with COND mice, T1DV mice had a significant difference in the relative abundance of the gut microbiota at the phylum level before FMT (Figure S17f), but these differences were gradually reduced after 7 and 14 days of FMT (Figures S17g and S17h). After FMT, the relative abundances of acetate-producing bacteria were significantly increased in T1DV mice, such as *Alistipes*, *Blautia*, *Ruminclostridium_9*, and *Roseburia* (Fig. 5b), resulting in a recovery of fecal acetate to normal level in T1DV mice (Fig. 5c). Interestingly, we found that FMT from COND mice significantly increased hippocampal SYP level in T1DV mice at both mRNA (Fig. 5d, P=0.05) and protein (Fig. 5e, f, P=0.02) levels. Immunofluorescence also illustrates a significantly increased intensity of SYP in hippocampal CA1 area after FMT (Fig. 5g, h, P=0.03). Taken together, these findings suggest that the gut microbiota can improve hippocampal SYP level via increasing acetate production in T1DV mice.

### Acetate improved hippocampal SYP level via vagus nerve stimulation

**Fig. 6:**
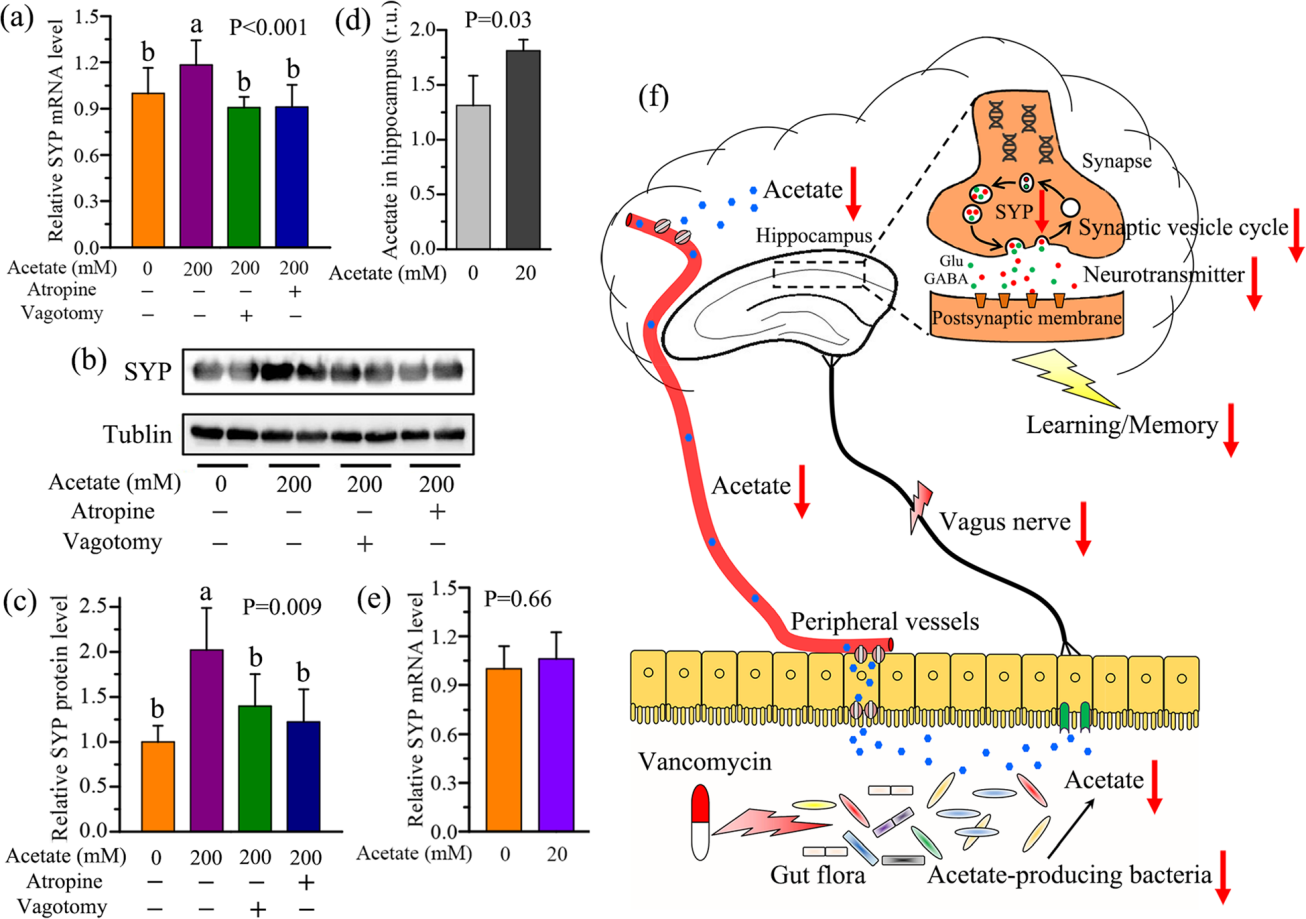
Acetate increases hippocampal synaptophysin level via the vagus nerve stimulation. **a** The effect of exogenous acetate supplement on the relative SYP mRNA expression level in the hippocampus of vancomycin-treated T1D (T1DV) mice after vagal inhibition by atropine or vagotomy. **b**, **c** Relative SYP protein level detected by Western blotting and the corresponding quantitative data in the hippocampus of acetate-fed T1DV mice after vagal inhibition by atropine or vagotomy (*n*=3 mice per group). **d**, **e** The changes in acetate and the relative SYP mRNA expression level in the hippocampus of T1DV mice after intracerebroventricular (ICV) injection of exogenous acetate. **f** Long-term acetate deficiency due to depletion of acetate-producing bacteria reduced hippocampal SYP level and accelerated cognitive impairment in T1D mice, which might be mediated by vagus nerve stimulation. Data are presented as mean±s.d. of *n*=6 mice per group. The difference between two groups was determined by two-tailed unpaired Student’s t test with Bonferroni correction. The differences among four groups were analyzed by one-way ANOVA with Bonferroni’s multiple comparisons test, and data with different lowercase codes are significantly different (P < 0.05)

The gut-brain axis can be mediated by vagus nerve stimulation [28]. To determine whether vagal inhibition or vagotomy suppresses acetate-induced increase in hippocampal SYP level of mice, atropine was used as a vagus nerve blocker. Acetate treatment significantly increased the hippocampal SYP level in T1DV mice at both mRNA (Fig. 6a) and protein (Fig. 6b, c) levels. After atropine treatment, however, T1DV mice with acetate infusion (T1DVA) still had a significant lower SYP level in the hippocampus (Fig. 6a–c), suggesting that atropine can abolish the ability of acetate to increase hippocampal SYP level. Considering the peripheral effect of atropine on the cardiac sinoatrial node, we carried out a bilateral vagotomy to further confirm the causal role of vagus nerve in the regulation of hippocampal SYP level. The results show that acetate-induced increase in SYP level in the hippocampus was suppressed in vagotomized T1DV mice at both mRNA (Fig. 6a) and protein (Fig. 6b, c) levels. To test whether the effect of acetate on hippocampal SYP level is centrally mediated, T1DV mice were administered with exogenous acetate by intracerebroventricular (ICV) injection. As expected, hippocampal acetate level was significantly increased after an ICV administration of acetate (Fig. 6d, P=0.03). However, central injection of acetate did not significantly alter the level of SYP in the hippocampus of T1DV mice (Fig. 6e, P=0.66). Therefore, our results suggest that acetate-induced increase in hippocampal SYP level is mediated through vagus nerve stimulation (Fig. 6f).

## Discussion

Cognitive decline is an important complication of diabetes, but the types of cognitive functions were influenced differently between two types of diabetes [4, 29]. In T2D patients, most affected cognitive functions mainly include memory, verbal fluency, attention, executive function, and processing speed [30, 31]. A meta-analysis involving 33 studies revealed that cognitive performances such as general intelligence, psychomotor efficiency, mental flexibility, and visual perception were mainly affected in T1D patients, but there was no significant impact on memory, motor speed, selective attention, and language [32]. However, significantly lower episodic short-term memory, mirrors visual attention, and psychomotor speed were detected in patients with long-term T1D (> 30 years) relative to age-matched controls [33]. Lacy et al. reported that severe hypoglycemia exposure caused worse cognitions including language, executive function, and episodic memory in older T1D patients [34]. Moreover, lower spatial working memory was observed in adolescents with T1D with acute hyperglycemia [35]. In our previous study, an impairment of spatial long-term learning and memory was found in both T1D mice [16] and rats [20]. We speculate that this type of cognitive dysfunction may also exist in older diabetic patients but lack of appropriate screening tests in clinical practice.

The gut microbiota has been implicated as a modulator of cognitive function [36] and as a novel therapeutic target for brain disorders [37]. The gut microbiota modified by polysaccharides [38] or intermittent-fasting [39] can improve diabetes-induced cognitive impairment. Vancomycin treatment decreased incidence of T1D in the NOD mice via increasing *Akkermansia muciniphila* [21], a bacterium having a protective effect on cognitive ability in a mouse model of Alzheimer’s disease [40]. To test whether vancomycin exposure might also improve T1D-associated cognitive decline, T1D mice were treated with vancomycin to modify the gut microbiota. Our results demonstrate that vancomycin exposure accelerated an impairment of spatial long-term learning and memory in T1D mice. The gut flora has also been reported to affect metabolic syndrome-related cognitive decline by regulating chronic inflammation [41]. However, our results suggest that hippocampal inflammatory response might not be responsible for vancomycin-driven cognitive dysfunction in T1D mice. The relative abundance of *Akkermansia muciniphila* was enriched after vancomycin treatment, but a significant reduction in acetate-producing bacteria also needed to be noticed. Long-term acetate deficiency resulted in an impaired learning and memory in both CON and T1D mice, while exogenous acetate supplement increased hippocampal SYP level and alleviated vancomycin-induced cognitive decline. In addition, FMT effectively recovered acetate-producing bacteria and the level of SYP in the hippocampus of T1D mice treated with vancomycin. Finally, we elucidated that acetate can regulate hippocampal SYP level via vagus nerve stimulation and improve cognitive functions in mice.

Acetate, one of the major SCFAs, is metabolized by the gut bacteria from dietary fiber fermentation and exerts a variety of physiological functions [42]. For example, acetate has been reported to suppress insulin-mediated fat accumulation [43], reduce appetite [44], modulate colonic serotonin secretion [45], protect against respiratory syncytial virus infection [46], and improve cardiovascular health [47]. However, acetate can also promote metabolic syndrome by the gut-microbiota-brain-β-cell axis [48]. In our study, we found that acetate increased hippocampal SYP level and thereby alleviated cognitive decline in mice. SYP, as a synaptic vesicle membrane protein, is not necessary for neurotransmitter release [49], but affects the efficiency of synaptic vesicle cycle [50]. The synaptic vesicle cycle plays an important role in the biological functions of presynaptic terminals and its disturbance would undermine cognitive ability [51]. Therefore, acetate may enhance the efficiency of synaptic vesicle cycle and thereby protect against cognitive impairment in mice. Another interesting finding from this study is that vagal inhibition or vagotomy can suppress the increase in hippocampal SYP level induced by acetate. The vagus nerve has been regarded as a modulator in the microbiota-gut-brain axis [28]. An earlier intriguing study has reported that acetate increased pancreatic insulin secretion and body weight gain by activating the parasympathetic nervous system in rats [48]. Goswami et al. found that vagal afferents may be implicated in suppression of food intake by acetate [52]. Herein we suggest the role of vagus nerve in mediating acetate-driven improvement in hippocampal SYP level and cognitive functions in T1D mice.

## Conclusions

In this study, we reveal that long-term acetate deficiency due to depletion of acetate-producing bacteria reduced hippocampal SYP level and accelerated cognitive impairment in T1D mice. This phenomenon might be mediated by the vagus nerve stimulation. Our study sheds new light on a novel function of microbiota metabolite acetate, but also suggests long-term acetate deficiency as a risk factor of cognitive decline. Moreover, our results propose a possible strategy for prevention and treatment of the adverse effect of lack of acetate-producing bacteria on cognitive functions.

## Materials and methods

### Animals

Male C57BL/6 mice aged 6 weeks (body weight = 20±1.5 g) were purchased from the SLAC Laboratory Animal Co., Ltd. (Shanghai, China), and housed in a specific pathogen-free (SPF) vivarium under a fully controlled condition (temperature, 22±1 °C; humidity, 55±5%; light/dark cycle, 12h/12h; lights on at 8:00 a.m.) at the Laboratory Animal Center of Wenzhou Medical University (WMU, Wenzhou, China). Mice were given free access to standard chow and tap water and acclimated to the experimental environment for at least 1 week. In this study, mouse chow and drinking water were prepared by irradiation and steam autoclave sterilizations before usage, respectively. All animal care and procedures were in accordance with the Guide for the Care and Use of Laboratory Animals and approved by the Institutional Animal Care and Use Committee of WMU (No.: wydw-2019-0332).

### Streptozocin-induced diabetic mice

To develop a type 1 diabetic (T1D) model, mice were fasted overnight, but allowed free access to water, prior to injection of streptozocin (STZ, Sigma-Aldrich). STZ solution was prepared in citrate buffer (0.5%, pH=4.3). Mice were given by intraperitoneal injection at dosage 40 mg/kg of body weight for five consecutive days. Meanwhile, control mice were injected with the same volume of sodium citrate. After 3 days of STZ injection, blood glucose level was measured from a tail nick using a handheld glucometer (ACCU-CHEK Active, Mannheim, Germany). After two consecutive measurements, mice were considered as T1D when glycemia exceeded 200 mg/dl. Additionally, body weight of mice was recorded with a digital balance (JY, Shanghai Minqiao Precise Science Co. Ltd., China).

### Vancomycin treatment

Mice were randomly divided into control and vancomycin groups. Before treatment, mice were adapted to the taste of vancomycin (Sigma Aldrich) by gavage for 3 days and then administered with vancomycin in their drinking water at a concentration of 0.5 g/l. The control mice were given water without any antibiotics until the end of vancomycin treatment.

### Vagotomy and atropine treatment

A group of mice were subjected to vagotomy or sham [53]. In brief, mice were anesthetized by intraperitoneal administration of isoflurane. Bilateral neck branches of the vagal nerve were split and cut. In the sham group, the vagal nerve of mice was only exposed but not cut. All mice were daily monitored and spontaneously restored for 1 week after surgery. Mice were treated with 0.1 mg/kg of atropine (Sigma) prepared in sterile saline by tail-vein injection 15 min prior to acetate treatment and every 30 min until 1 h after acetate administration. The dose and time of atropine treatment were referred to the previous study [48]. In addition, mice were treated with sterile saline as a control group.

### Acetate administration

Mice were treated with acetate by three different delivery methods. In the first study, acetate was administered to mice in their drinking water at a concentration of 120 mM (pH=7.35) for 7 weeks. In the second study, normal control, atropine-treated, or vagotomized mice were treated with 100 μl of acetate solution prepared in sterile saline (200 mM, pH=7.71) via tail-vein injection. In the third study, we administered 1 μl of acetate (20 mM, pH=7.02) into the third ventricle of mice by intracerebroventricular injection using micro-syringes at a rate of 0.2 μl/min, and the cannula was kept for 2 min. Moreover, control mice were injected the same volume of sterile saline. To match the pH value, drinking water and sterile saline were neutralized by using sodium bicarbonate. The first and second studies were continued for 1 h. After acetate administration, mice were sacrificed by rapid decapitation, and the hippocampus tissue was collected, frozen in liquid nitrogen, and stored at −80 °C until analysis.

### Fecal microbiota transplantation (FMT)

In the current study, co-housing with fecal gavage was carried out to investigate the effect of the gut microbiota on hippocampal SYP level. After 7 weeks of vancomycin treatment, mice were given autoclaved drinking water without any antibiotics. For the co-housing feeding, two normal control mice were co-housed with four vancomycin-treated T1D (T1DV) mice (6 mice per cage). In addition, another group of six normal control mice were used as fecal donor (COND) mice. Fresh fecal sample from COND mice was collected and mixed with autoclaved sterile PBS (1:20, w/v) every day during FMT period. The mixture was vortexed vigorously for 30 s and centrifuged at 1000*g* for 5 min at 4 °C. Afterward, 100 μl of the supernatant was transplanted to T1DV mice by intragastric administration once daily for 14 days (Fig. 5a). All FMT procedures were carried out under SPF condition and finished within 10 min.

### Morris water maze (MWM) test

The MWM test was used to assess learning and memory ability in mice. Briefly, the MWM test was carried out in a circular pool filled with opaque water (26 ± 1 °C). Moreover, the escape platform was submerged 1 cm below water surface. The MWM test included two periods for 5 days, training and test. During a consecutive 4 days of training, mice were guided to reach the escape platform by the operator, when mice cannot find the platform within 60s. In the fifth day, the escape platform was removed from the circular pool and then the trained mice were subjected to a 90-s test trial. The overhead camera and computer system were utilized to record their behavior. The escape latency during the training phase and swimming length and time in the target quadrant and the number of crossings over the original platform location during the test phase were calculated by using the Viewer-2 software (Biobserve GmbH, Bonn, Germany).

### Sample collection and preparation

Fecal pellets of mice were freshly collected before sacrifice in metabolic cages and immediately frozen at −80 °C until use. Mice were sacrificed by rapid decapitation, and cecal contents and tissue, colonic tissue, and the hippocampus were collected, frozen in liquid nitrogen promptly and kept at −80 °C until analysis. Fecal pellets and cecal contents were divided into two parts for metabolomics and microbial analyses, respectively. In addition, blood sample was collected and centrifuged at 3000*g* for 15 min at 4 °C to separate serum and stored at − 80 °C until use.

Serum sample (200 μl) was thawed and mixed with 250 μl of phosphate buffer (0.2 mM Na_2_HPO_4_/NaH_2_PO_4_, pH=7.4) and 50 μl of D_2_O for signal locking. The diluted serum sample was vortexed for 10 s, centrifuged at 12,000*g* for 15 min at 4 °C, and then 500 μl of supernatant was transferred into a 5-mm NMR tube for metabolomics analysis. Fecal pellets and cecal contents (0.1 g) were thawed and homogenized in 0.5 ml of phosphate buffer in centrifuge tubes, respectively. The mixture was extracted by ultrasonic-associated extraction for 10 min and centrifuged at 5000*g* for 15 min at 4 °C. Then, 400 μl of supernatant was mixed with 100 μl of D_2_O containing 0.05% of sodium trimethylsilyl propionate-d_4_ (TSP, 0.42 mM) and transferred into an NMR tube for analysis. For colonic and hippocampal tissues, approximately 0.1 g of sample was weighed into a centrifuge tube and added with 4 ml/g of ice-cold methanol and 0.85 ml/g of ice-cold water. The tissue sample was then homogenized using a handheld electric homogenizer (FLUKO, Shanghai, China) and mixed with 2 ml/g of ice-cold chloroform and ice-cold water. After blending, the mixture was vortexed vigorously, stood on ice for 15 min, and centrifuged at 10,000*g* for 15 min at 4 °C. The supernatant was transferred into a new centrifuge tube and lyophilized for 24h. Finally, the lyophilized powder was redissolved in 500 μl of D_2_O (0.05% TSP) and transferred into an NMR tube for metabolomics analysis.

### NMR-based metabolomics analysis

^1^H NMR spectra were acquired by using a Bruker AVANCE III 600 MHz NMR spectrometer with a 5-mm TXI probe (Bruker BioSpin, Rheinstetten, Germany). A standard single-pulse sequence with water signal pre-saturation (ZGPR) was used to measure metabolic profiles of extracts from fecal pellets, cecal contents and tissue, colonic tissue, and the hippocampus. The main acquisition parameters were set as follows: data points, 256 K; spectral width, 12,000 Hz; acquisition time, 2.66 s/scan; and relaxation delay, 4 s. For serum sample, a CPMG pulse sequence with a fixed receiver gain value was employed to minimize the line-broadening effect of protein or lipid. The main parameters included data points, 256 K; spectral width, 12,335.5 Hz; acquisition time, 2.66 s/scan; and relaxation delay, 4 s.

All ^1^H NMR spectra were manually corrected for baseline and phase using Topspin 3.0 software (Bruker, Rheinstetten, Germany). The spectra of extracts were referenced to TSP peak at 0 ppm, while the spectra of serum were referenced to the anomeric signal of α-glucose at 5.23 ppm. Subsequently, an *icoshift* procedure was applied to align NMR spectra in MATLAB software (R2012a, The Mathworks Inc., Natick, MA, USA) [54]. The NMR spectra from 0.0 to 9.0 ppm excluding residual water signals (4.7–5.2 ppm) were subdivided and integrated to binning data with a size of 0.01 ppm for multivariate analysis.

Metabolite signals were assigned using Chenomx NMR suite 7.0 (Chenomx Inc., Edmonton, Canada) and human metabolome database [55]. To further confirm tentative identifications, a ^13^C-^1^H heteronuclear single quantum coherence experiment (HSQC) was conducted to analyze representative samples. Typical ^1^H NMR spectra obtained from fecal pellets, cecal contents, cecal tissue, colonic tissue, serum, and the hippocampus were illustrated in Figures S8a-S8f, respectively. In addition, the peak area of specific metabolite was manually integrated, and its relative concentration was quantified on the basis of its peak area by reference to TSP peak area.

### Microbial DNA extraction

Fresh fecal pellets and cecum content samples were collected from mice in sterile containers and immediately kept at −80 °C until use. The microbial DNA was extracted by using the stool DNA isolation kit (TianGen, Beijing, China) according to the manufacturer’s instructions with modifications. Briefly, aliquots of approximately 100-mg samples were mixed with 1 ml of lysis buffer, homogenized and vortex vigorously for 1 min. The mixtures were incubated at 95 °C for 5 min and centrifuged at 13,000*g* for 5 min, and then, the DNA pellets were dissolved in 120 μl of Tris-acetate-EDTA (TAE) buffer. DNA integrity and size were detected by 1.0% agarose gel electrophoresis (AGE) at 100 V, and the concentration of DNA was measured using a NanoDrop 2000 spectrophotometer (Thermo Scientific, USA). In addition, blank extracted samples in which the DNA extraction process was followed with sterile water instead of biological samples were used as the negative control to determine background microbial signal.

### 16S rRNA gene sequencing and analysis

The V4 region of the bacterial 16S rRNA gene was amplified with the barcoded primers 515F (5′-GTG CCA GCM GCC GCG GTA A-3′) and 806R (5′-GGA CTA CHV GGG TWT CTA AT-3′). PCR was conducted with 15 μl of Phusion High-Fidelity PCR Master Mix with GC buffer (NEB, Ipswich, MA, USA), 3 μl of forward and reverse primers, 10 ng of template DNA, and 2 μl of sterile water. PCR amplification was performed using a Bio-Rad T100 System (Bio-Rad, Hercules, CA, USA) under the following program: initial denaturation at 98 °C for 1 min, 30 cycles (denaturation at 98 °C for 10 s, annealing at 50 °C for 30 s, and elongation at 72 °C for 30 s), and final elongation at 72 °C for 5 min. PCR products were detected by 2% AGE, purified with a QIAquick gel extraction kit (Qiagen, Germany), and then sequenced on an Illumina HiSeq2500 PE250 sequencer (San Diego, USA) at Novogene (Beijing, China).

All sequences were assigned to different samples according to their unique barcodes, and then raw tags were generated through merging paired-end reads by using FLASH software (v1.2.7). Raw tags were filtered and developed into clean tags according to QIIME (v1.7.0) analysis. Clean tags were aligned to Gold database, and non-chimera clean tags were detected as effective tags by using UCHIME algorithm (v7.0.1001). Subsequently, effective tags were clustered into operational taxonomic units (OTUs) using UPARSE pipeline (v7.0.1001) with a similarity threshold of 97% [56]. Taxonomy assignment was conducted in accordance to the Mothur method and SILVA database [57]. The alpha-and beta-diversity of the gut microbiome were analyzed by QIIME software (v1.7.0) [58] and R software (v2.15.3).

### Metagenomic sequencing and analysis

In this study, sequencing libraries were generated with approximately 1 μg of DNA per cecum content sample using NEBNext® Ultra™ DNA Library Prep Kit for Illumina (NEB, USA), and index codes were used to attribute sequences to each sample. In brief, DNA sample was fragmented into 350 bp by using a sonication method, and DNA fragments were end-polished, A-tailed and ligated with the full-length adaptor for Illumina sequencing and then amplified by PCR. PCR products were purified by using the AMPure XP system and libraries were prepared on a cBot Cluster Generation System based on the manufacturer’s instructions. Finally, the library preparations were analyzed on an Illumina HiSeq2500 PE150 sequencer at Novogene (Beijing, China) and paired-end reads were generated.

Sequencing adapters and low-quality reads, including sequences with more than 40 bases and with quality score lower than 38 or with N bases more than 10, were filtered by using Readfq (v8, https://github.com/cjfields/readfq). High-quality reads were assembled to generate a number of scaffolds using SOAPdenovo software (v2.04, http://soap.genomics.org.cn/soapdenovo.html). MetaGeneMark (v2.10, http://topaz.gatech.edu/GeneMark/) was employed to predict open reading frames (ORFs), and redundancy was removed using CD-HIT software (v4.5.8, http://www.bioinformatics.org/cd-hit/). DIAMOND (v0.9.9.110, https://github.com/bbuchfink/diamond/) was used to analyze unigene sequence files from NR database at NCBI (https://www.ncbi.nlm.nih.gov/) with the Basic Local Alignment Search Tool (BLAST). The lowest common ancestor (LCA) algorithm was performed to assign ORF alignments into taxonomic groups. The functional profile of KEGG orthology (KO) was predicted based on metagenome data with PICRUSt software [59]. Subsequently, the predicted KO abundances were categorized as levels 1-3 into KEGG pathways.

### Real-time qPCR analysis

Total RNA in the hippocampus was isolated using TRIzol reagent (Invitrogen, CA, USA). The quantity and quality of RNA were determined with a NanoDrop spectrometer. Reverse transcription was performed with PrimeScript^TM^ RT reagent kit (TaKaRa, RR037A, Tokyo, Japan). Primer pairs were used to detect different target gene transcripts, as shown in Table S1. Quantitative mRNA analysis was conducted by a StepOnePlus Real-Time PCR System (Life Technologies) using the SYBR Green fluorescence system according to the manufacturer’s instructions. Then, the level of mRNA expression was quantified with the ΔΔCT method using GAPDH as a calibrator gene. All samples were analyzed at least in triplicates, and target gene expression was presented as relative expression level.

### Western blot analysis

Proteins were extracted from hippocampal tissue using RIPA buffer containing proteinase (Complete, Roche) and phosphatase inhibitors (1 mM β-glycerophosphate, 10 mM NaF, and 0.1 mM Na_3_VO_4_). Total protein was determined using a BCA protein assay kit, run on polyacrylamide gel, and transferred on to a PVDF membrane (Millipore, Billeria). The membrane was blocked with 10% milk in PBS for 1 h at room temperature and incubated overnight at 4 °C with synaptophysin (SYP) (1:1000, cell signal technology) and anti-α-tublin (1:1000, Abcam) antibodies. After washing five times with TBST, the membrane was incubated with anti-rabbit IgG-HRP secondary antibody (1:10000, cell signal technology) at room temperature for 1 h. Proteins were detected with a chemiluminescence (ECL) kit (Millipore, Billeria). Then, the relative density of protein bands was scanned using an LAS 4000 Fujifilm imaging system (Fujifilm, Tokyo, Japan) and analyzed by Quantity-One software (Bio-Rad Hercules, CA, USA).

### Immunofluorescence

Mice were anaesthetized with isoflurane and perfused with 4% paraformaldehyde prepared in PBS (0.1 M, pH=7.5). Brain and pancreas tissues were isolated, fixed in 10% neutral formalin, dehydrated in a graded series of ethanol, embedded in paraffin, and cut into 5-μm slices using a slicing machine (Leica, Germany). For immunofluorescence, slides after deparaffinization and rehydration were placed in citric acid buffer (10 mM, pH=6.5) within a decloaking chamber at 120 °C for 5 min and cooled at room temperature for 1 h. Then, slides were washed two times in PBS for 5 min, treated with 3% H_2_O_2_ for 30 min and blocked with 5% BSA for 30 min at 37 °C. Sections were incubated with primary antibody SYP (1:200, cell signal technology) at 4 °C overnight. After triple washing with PBS (5 min/time), sections were incubated with anti-rabbit IgG-HRP secondary antibody (1:500, cell signal technology) at 37 °C for 1 h. Sections were washed in PBS (5 min/time) and added with DAPI (200 ng/ml) for 2 min at room temperature. Pancreas sections were incubated with primary antibody anti-insulin (Abcam, ab181547) and anti-glucagon (Abcam, ab10988) overnight at 4 °C and then incubated with secondary antibody (1:500, anti-rabbit IgG-HRP) at 37 °C for 1 h. Finally, the images were captured by using a Nikon ECLIPSE Ti microscope and processed by the ImageJ software.

### Immunohistochemistry

The inflammatory (TNF-α) and anti-inflammatory (IL-10) cytokines in the hippocampus of mice were detected by immunohistochemistry (IHC) staining. In brief, 5 μm paraffin-embedded brain slices were incubated with the first primary antibodies including rabbit anti-IL-10 (1:100, Proteintech, Wuhan, China) and rabbit anti-TNF-α (1:50, Abcam, Cambridge, UK) overnight at 4 °C. Then, sections were incubated with goat anti-rabbit immunoglobulin (IgG)-horseradish peroxidase (HRP) secondary antibody (1:200, Affinity, Jiangsu, China) at 37 °C for 1 h. Finally, the images were acquired by using a Nikon DS-Ri2 light microscope.

### Data analysis and statistics

Mice were randomly assigned to experimental procedures, including animal grouping, antibiotic treatment, and sample collection. All analyses in this study were performed by masking animal labels. Data were pareto-scaled and log-transformed prior to multivariable analysis. Principal component analysis (PCA) was employed to obtain an overview of metabolic pattern changes among different groups using SIMCA-P+ 12.0 software (Umetrics AB, Umeå, Sweden). Furthermore, partial least squares-discriminate analysis (PLS-DA) was used to examine the difference between two groups using SIMCA software, and important metabolites responsible for classification were identified by S-plot. Beta-diversity of the gut microbiota was analyzed by principal coordinate analysis (PCoA) based on Bray-Curtis distance using R software (version 2.15.3).

The difference between two groups was determined by two-tailed unpaired Student’s t test with Bonferroni correction in SAS 9.4 (SAS Institute Inc, Cary, NC). The difference among three or four groups was analyzed by ANOVA with Bonferroni correction in SAS 9.4. The difference was significant when *P*<0.05, and different lowercase letters in the figure indicate statistically significant differences. Additionally, the escape latency from the MWM test was analyzed with a repeated measure ANOVA in SAS 9.4 (PROC GLM procedure). The relationship between different variables was analyzed by Pearson’s correlation and its P value was also calculated using MATLAB software (“CORR” function, R2012a, The MathWorks, Inc.). Correlation network was visualized using Cytoscape software (v3.6.0) [60].

## Supplementary Information

**Additional file 1: Table S1.** Specific primer pairs for RT-qPCR analysis. **Figure S1.** Streptozocin-induced type 1 diabetic (T1D) mouse model. **Figure S2.** The Morris water maze (MWM) test. **Figure S3.** Hippocampal inflammation analysis. **Figure S4.** The Morris water maze (MWM) test. **Figure S5.** Vancomycin exposure alters the gut microbiota in type 1 diabetic (T1D) mice. **Figure S6.** Vancomycin exposure alters the gut microbiota patterns in normal healthy mice. **Figure S7.** Vancomycin exposure alters the gut microbiota composition in normal healthy mice. **Figure S8.** NMR-based metabolomic profiling. **Figure S9.** Unsupervised metabolic pattern analysis. **Figure S10.** Supervised metabolic pattern analysis. **Figure S11.** Vancomycin exposure decreases the level of acetate in type 1 diabetic (T1D) mice. **Figure S12.** Vancomycin exposure decreases the levels of butyrate and propionate in type 1 diabetic (T1D) mice. **Figure S13.** The Morris water maze (MWM) test. **Figure S14.** The effects of exogenous butyrate and propionate supplements on learning and memory in vancomycin-treated T1D (T1DV) mice. **Figure S15.** Metabolomics data analysis. **Figure S16.** The effects of exogenous butyrate and propionate supplements on hippocampal SYP level in vancomycin-treated T1D (T1DV) mice. **Figure S17.** Fecal microbiota transplant (FMT) reshapes the gut microbiota in vancomycin-treated T1D mice.

## Data Availability

All data used in this study are present in the main text and supplementary materials. Metabolomics and microbiomics data have been made publicly available in Figshare: NMR-based metabolomics data (10.6084/m9.figshare.10063058; 10.6084/m9.figshare.14281679.v1), 16S rRNA gene sequencing data (10.6084/m9.figshare.10075214; 10.6084/m9.figshare.10075412; 10.6084/m9.figshare.14279123.v1), and metagenomic sequencing data (10.6084/m9.figshare.10101377.v2). Scripts are available in Github (https://github.com/hongzheng1985/acetate-cognition). Additional data and materials can also be requested from first author or corresponding author.

## Abbreviations

CON: Normal control
COND: Healthy donor
FMT: Fecal microbiota transplant
NMR: Nuclear magnetic resonance
SCFA: Short-chain fatty acid
STZ: Streptozotocin
SYP: Synaptophysin
T1D: Type 1 diabetes mellitus
T1DV: Vancomycin-treated type 1 diabetes
T1DVA: Vancomycin-treated type 1 diabetes fed with acetate
T1DVB: Vancomycin-treated type 1 diabetes fed with butyrate
T1DVP: Vancomycin-treated type 1 diabetes fed with propionate
