# Correction to: Depletion of acetate-producing bacteria from the gut microbiota facilitates cognitive impairment through the gut-brain neural mechanism in diabetic mice

**DOI:** 10.1186/s40168-021-01127-5

**Published:** 2021-07-28

**Authors:** Hong Zheng, Pengtao Xu, Qiaoying Jiang, Qingqing Xu, Yafei Zheng, Junjie Yan, Hui Ji, Jie Ning, Xi Zhang, Chen Li, Limin Zhang, Yuping Li, Xiaokun Li, Weihong Song, Hongchang Gao

**Affiliations:** 1 Institute of Metabonomics & Medical NMR, School of Pharmaceutical Sciences, Wenzhou Medical University, Wenzhou, 325035 China; 2 Department of Pulmonary and Critical Care Medicine, The First Affiliated Hospital of Wenzhou Medical University, Wenzhou, 325015 China; 3 Institute of Aging, School of Mental Health, Wenzhou Medical University, Wenzhou, 325035 China; 4 State Key Laboratory of Magnetic Resonance and Atomic and Molecular Physics, Wuhan Institute of Physics and Mathematics, Chinese Academy of Sciences, Wuhan, 430070 China

**Correction to: Microbiome 9, 145 (2021)**

**https://doi.org/10.1186/s40168-021-01088-9**

Following the publication of the original article [1], the author realized that they made a translation mistake between Chinese character and English name for one of the authors’ name. Xiaokui Li should be revised to Xiaokun Li.

The original article has been updated.
