# Supplementary material for: Depletion of acetate-producing bacteria from the gut microbiota facilitates cognitive impairment through the gut-brain neural mechanism in diabetic mice
Source: Microbiome. 2021 Jun 25;9:145. doi: 10.1186/s40168-021-01088-9 (PMC8235853; doi:10.1186/s40168-021-01088-9)
Supplement: Supplementary file 2 — Additional file 1: Table S1. Specific primer pairs for RT-qPCR analysis. Figure S1. Streptozocin-induced type 1 diabetic (T1D) mouse model. Figure S2. The Morris water maze (MWM) test. Figure S3. Hippocampal inflammation analysis. Figure S4. The Morris water maze (MWM) test. Figure S5. Vancomycin exposure alters the gut microbiota in type 1 diabetic (T1D) mice. Figure S6. Vancomycin exposure alters the gut microbiota patterns in normal healthy mice. Figure S7. Vancomycin exposure alters the gut microbiota composition in normal healthy mice. Figure S8. NMR-based metabolomic profiling. Figure S9. Unsupervised metabolic pattern analysis. Figure S10. Supervised metabolic pattern analysis. Figure S11. Vancomycin exposure decreases the level of acetate in type 1 diabetic (T1D) mice. Figure S12. Vancomycin exposure decreases the levels of butyrate and propionate in type 1 diabetic (T1D) mice. Figure S13. The Morris water maze (MWM) test. Figure S14. The effects of exogenous butyrate and propionate supplements on learning and memory in vancomycin-treated T1D (T1DV) mice. Figure S15. Metabolomics data analysis. Figure S16. The effects of exogenous butyrate and propionate supplements on hippocampal SYP level in vancomycin-treated T1D (T1DV) mice. Figure S17. Fecal microbiota transplant (FMT) reshapes the gut microbiota in vancomycin-treated T1D mice. [file 40168_2021_1088_MOESM2_ESM.docx]

Depletion of acetate-producing bacteria from the gut microbiota facilitates cognitive impairment through the gut-brain neural mechanism in diabetic mice

Hong Zheng^1,2,3^, Pengtao Xu^1^, Qiaoying Jiang^1^, Qingqing Xu^1^, Yafei Zheng^1^, Junjie Yan^1^, Hui Ji^1^, Jie Ning^1^, Xi Zhang^1^, Chen Li^1^, Limin Zhang^4^, Yuping Li^2^, Xiaokui Li^1^, Weihong Song^3,*^, and Hongchang Gao^1,2,3,*^

^1^Institute of Metabonomics & Medical NMR, School of Pharmaceutical Sciences, Wenzhou Medical University, Wenzhou 325035, China; [123zhenghong321@163.com](mailto:123zhenghong321@163.com) (HZ); [xupt92@163.com](mailto:xupt92@163.com) (PTX); [jqying0580@126.com](mailto:jqying0580@126.com) (QYJ); [xuqingqing199701@163.com](mailto:xuqingqing199701@163.com) (QQX); [zhengyaf153@163.com](mailto:zhengyaf153@163.com) (YFZ); [yanjunjie0026@163.com](mailto:yanjunjie0026@163.com) (JJY); [jh1290487836@163.com](mailto:jh1290487836@163.com) (HJ); [m18826108312@163.com](mailto:m18826108312@163.com) (JN); [xizhang580@126.com](mailto:xizhang580@126.com) (XZ); [lichen2zh@163.com](mailto:lichen2zh@163.com) (CL); [xiaokunli@wmu.edu.cn](mailto:xiaokunli@wmu.edu.cn) (XKL)

^2^Department of Pulmonary and Critical Care Medicine, The First Affiliated Hospital of Wenzhou Medical University, Wenzhou 325015, China; [wzliyp@163.com](mailto:wzliyp@163.com) (YPL)

^3^Institute of Aging, School of Mental Health, Wenzhou Medical University, Wenzhou 325035, China

^4^State Key Laboratory of Magnetic Resonance and Atomic and Molecular Physics, Wuhan Institute of Physics and Mathematics, Chinese Academy of Sciences, Wuhan 430070, China; [zhanglm@wipm.ac.cn](mailto:zhanglm@wipm.ac.cn) (LMZ)

*Corresponding author. Email: [weihong@wmu.edu.cn](mailto:weihong@wmu.edu.cn) (WS); [gaohc27@wmu.edu.cn](mailto:gaohc27@wmu.edu.cn) (HCG).

**Table S1.** Specific primer pairs for RT-qPCR analysis.

| Gene | 5’-3’ primer sequence |
| --- | --- |
| SYP | F: AAGTATTGTGGTTTGGAG  R: GTAGATTCTGTCTTCTGATT |
| c-Fos | F: TTCTCATAGCACTAACTAATCT  R: CAGGAACACAGTAGGTATT |
| Arc | F: CAAGAGAGTGTGGCTATC  R: TTGAGGTAAGATGGTATGG |
| EGR | F: ACTCTGCTGTGACATTAGG  R: AACGGAACAACACTCTGA |
| GAPDH | F: GCTCTCTGCTCCTCCTGTTC  R: TTCCCGTTCTCAGCCTTGAC |


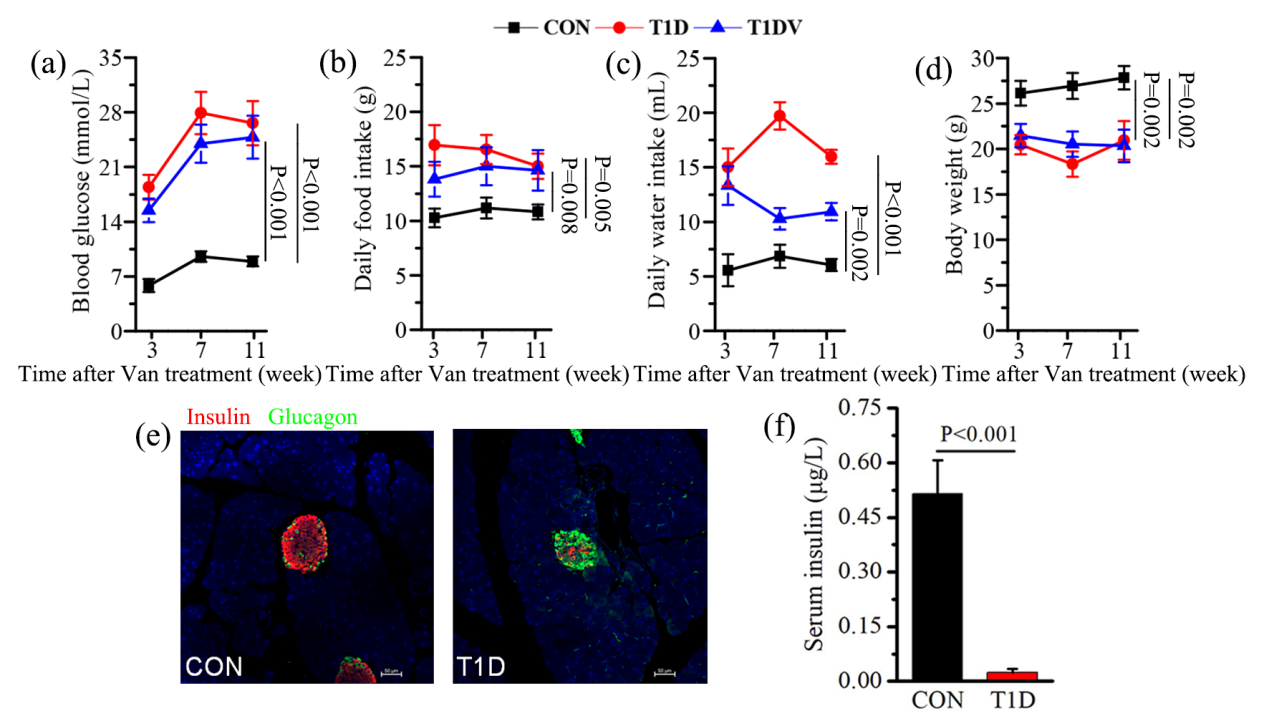


**Figure S1.** Streptozocin-induced type 1 diabetic (T1D) mouse model. The changes in **(a)** blood glucose level, **(b)** daily food intake, **(c)** daily water intake and **(d)** body weight in normal control (CON), T1D and vancomycin-treated T1D (T1DV) mice at 3, 7 and 11 weeks. **(e)** Visualization of α-and β-cells in pancreas of CON and T1D mice using fluorescence microscopy (scale bar = 50 μm). **(f)** The change in fasting insulin level between CON and T1D mice. Data are presented as mean±s.d. of *n*=6 mice per group. Time-series data were analyzed by repeated measures one-way ANOVA followed by Bonferroni’s multiple comparisons test. The difference between two groups was determined by two-tailed unpaired student’s t test with Bonferroni correction.


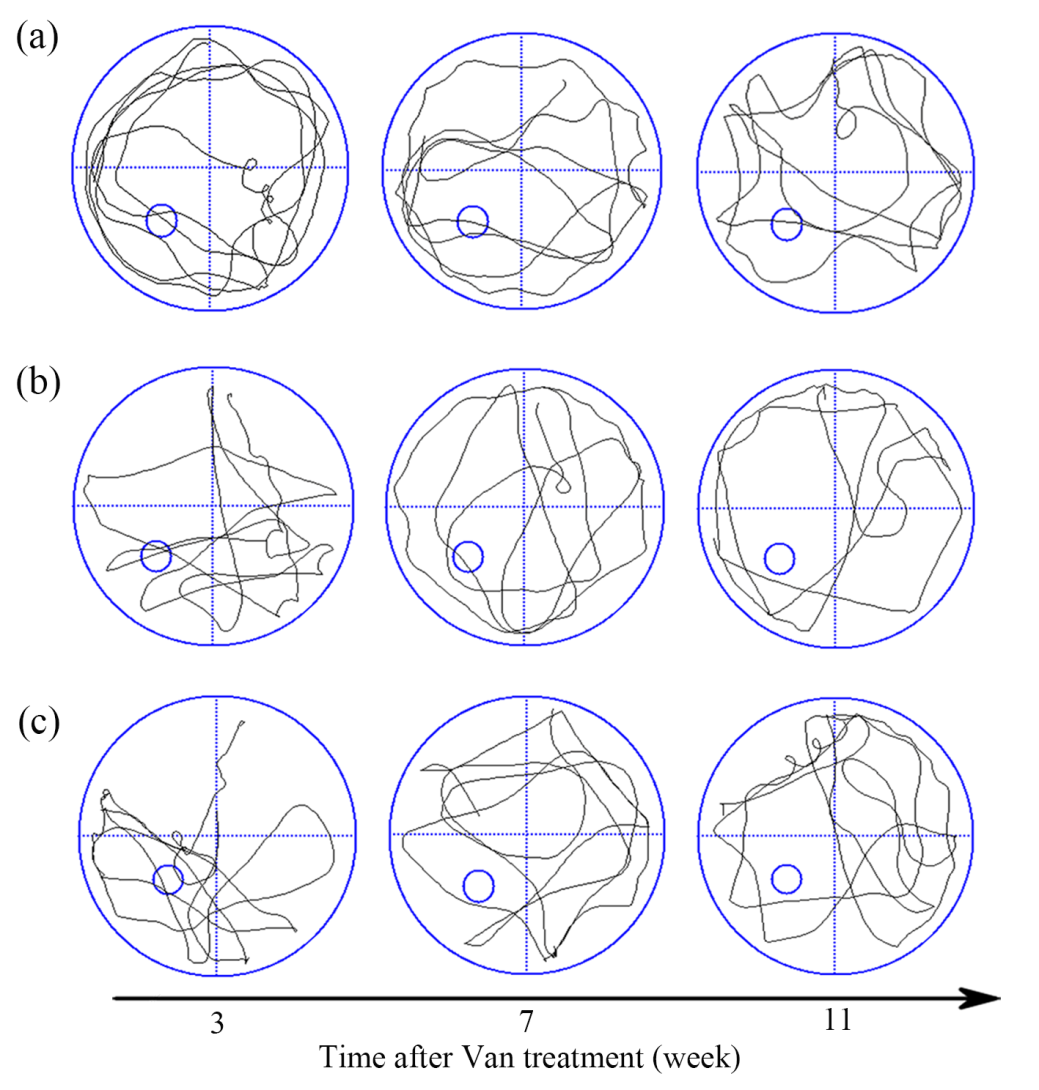


**Figure S2.** The Morris water maze (MWM) test. After a 1-week acclimation, mice were injected with streptozocin for 5 consecutive days to develop type 1 diabetic (T1D) mice and then administered with vancomycin (Van). The MWM test was used to evaluate learning and memory ability in T1D mice at 3, 7 and 11 weeks after Van treatment. This figure illustrates swimming path during the test period in **(a)** normal control (CON), **(b)** T1D and **(c)** Van-treated T1D (T1DV) mice at 3, 7 and 11 weeks.


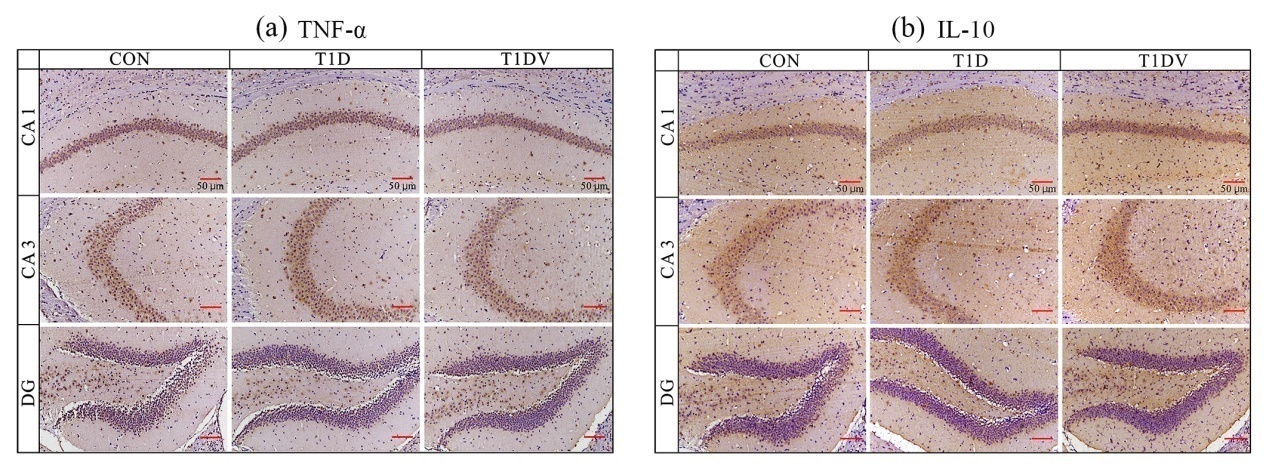


**Figure S3.** Hippocampal inflammation analysis. **(a)** Inflammatory cytokine, TNF-α, and **(b)** anti-inflammatory cytokine, IL-10, were detected in hippocampus of normal control (CON), type 1 diabetic (T1D) and vancomycin-treated T1D (T1DV) mice at 7 weeks by using immunohistochemical staining (*n*=3 mice per group). Scale bar = 50 μm.


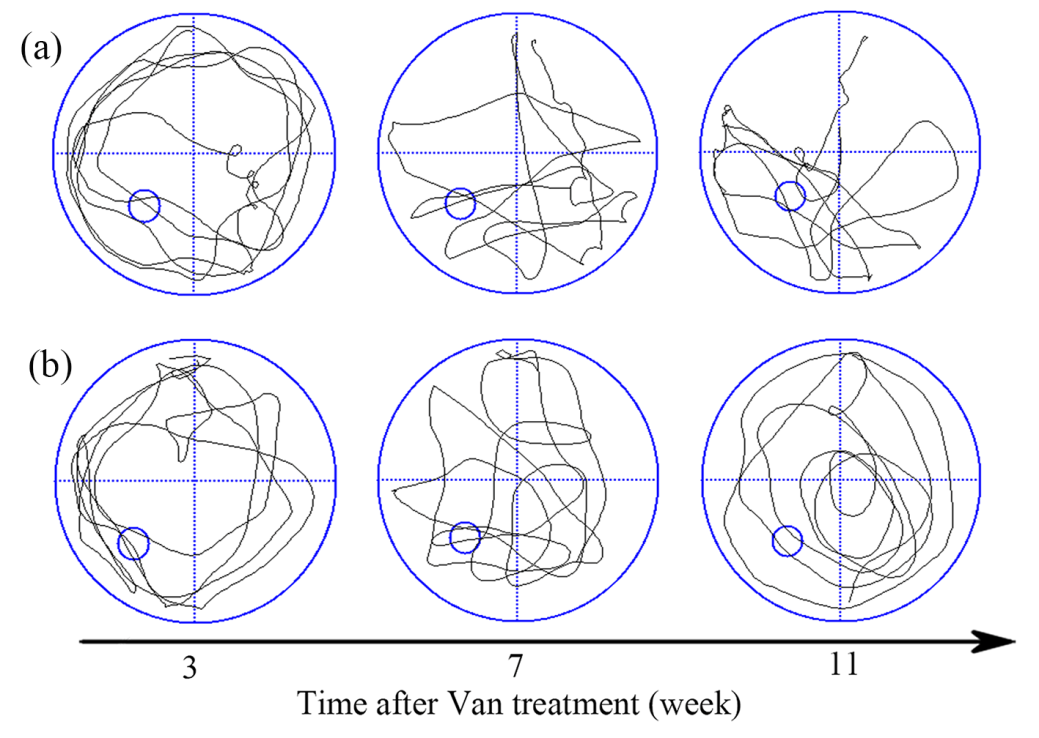


**Figure S4.** The Morris water maze (MWM) test. After a 1-week acclimation, normal healthy mice were treated with vancomycin (Van) and then their learning and memory ability was assessed at 3, 7 and 11 weeks after Van treatment. This figure shows swimming path during the test period in **(a)** normal control (CON) and **(b)** Van-treated CON (CONV) mice at 3, 7 and 11 weeks.


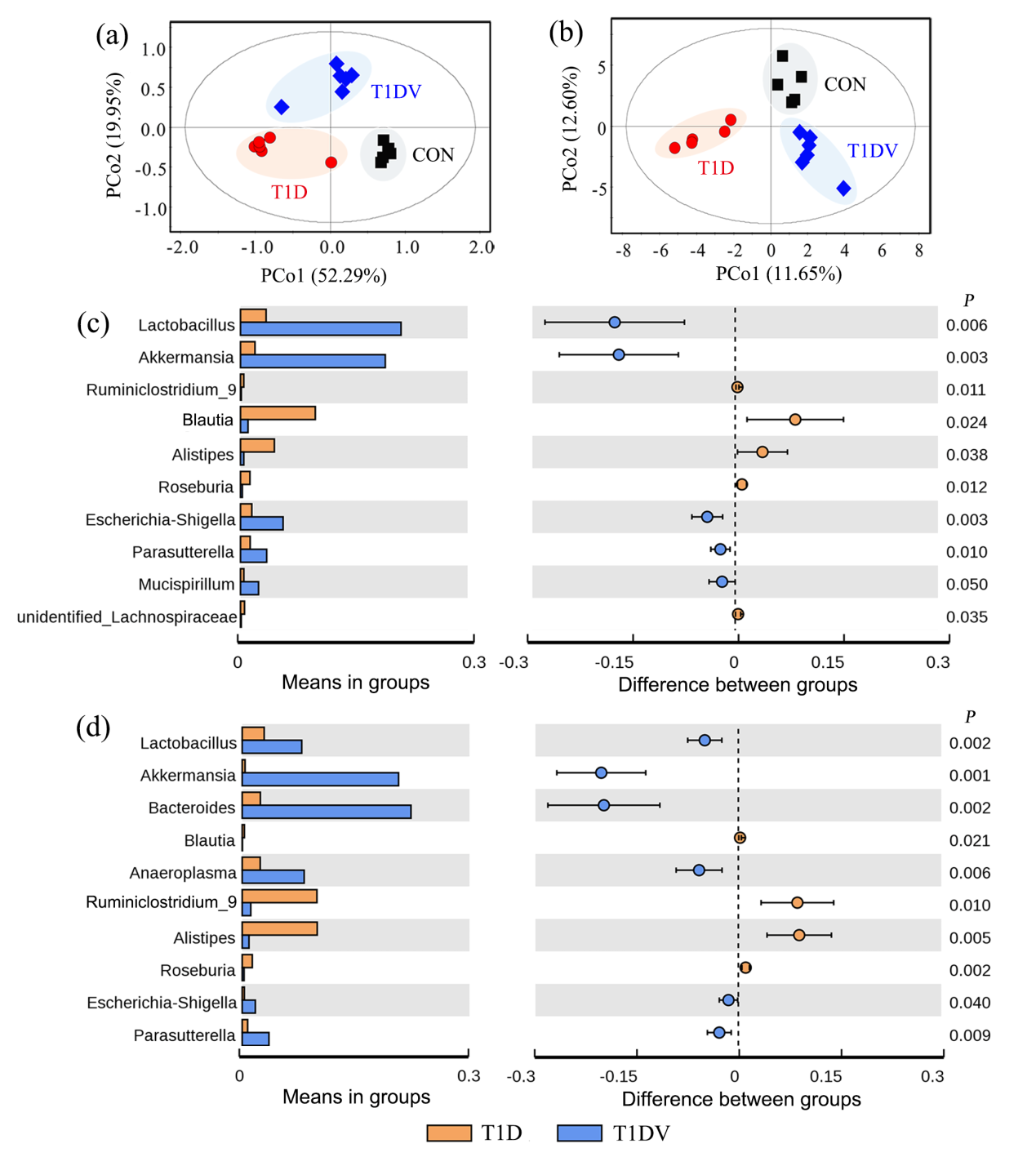


**Figure S5.** Vancomycin exposure alters the gut microbiota in type 1 diabetic (T1D) mice. PCoA-based classification using the gut microbiome at the genus level in caecum contents of normal control (CON), T1D and vancomycin-treated T1D (T1DV) mice at **(a)** 3 and **(b)** 11 weeks (*n*=5-6 mice per group). Top 10 microbes that significantly altered between T1D and T1DV mice at **(c)** 3 and **(d)** 11 weeks. The difference between two groups was determined by two-tailed unpaired student’s t test with Bonferroni correction.


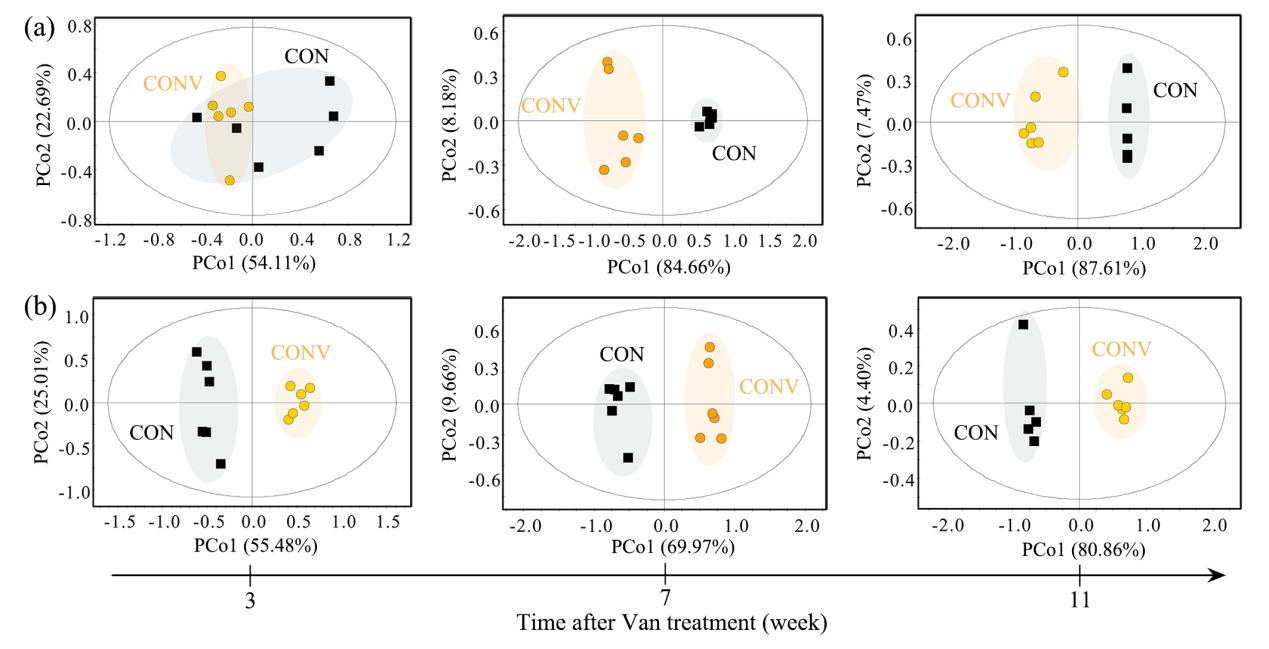


**Figure S6.** Vancomycin exposure alters the gut microbiota patterns in normal healthy mice. PCoA-based classification using the gut microbiome at both the **(a)** phylum and **(b)** genus levels in caecum contents of normal control (CON) and vancomycin-treated CON (CONV) mice at 3, 7 and 11 weeks (*n*=6 mice per group).


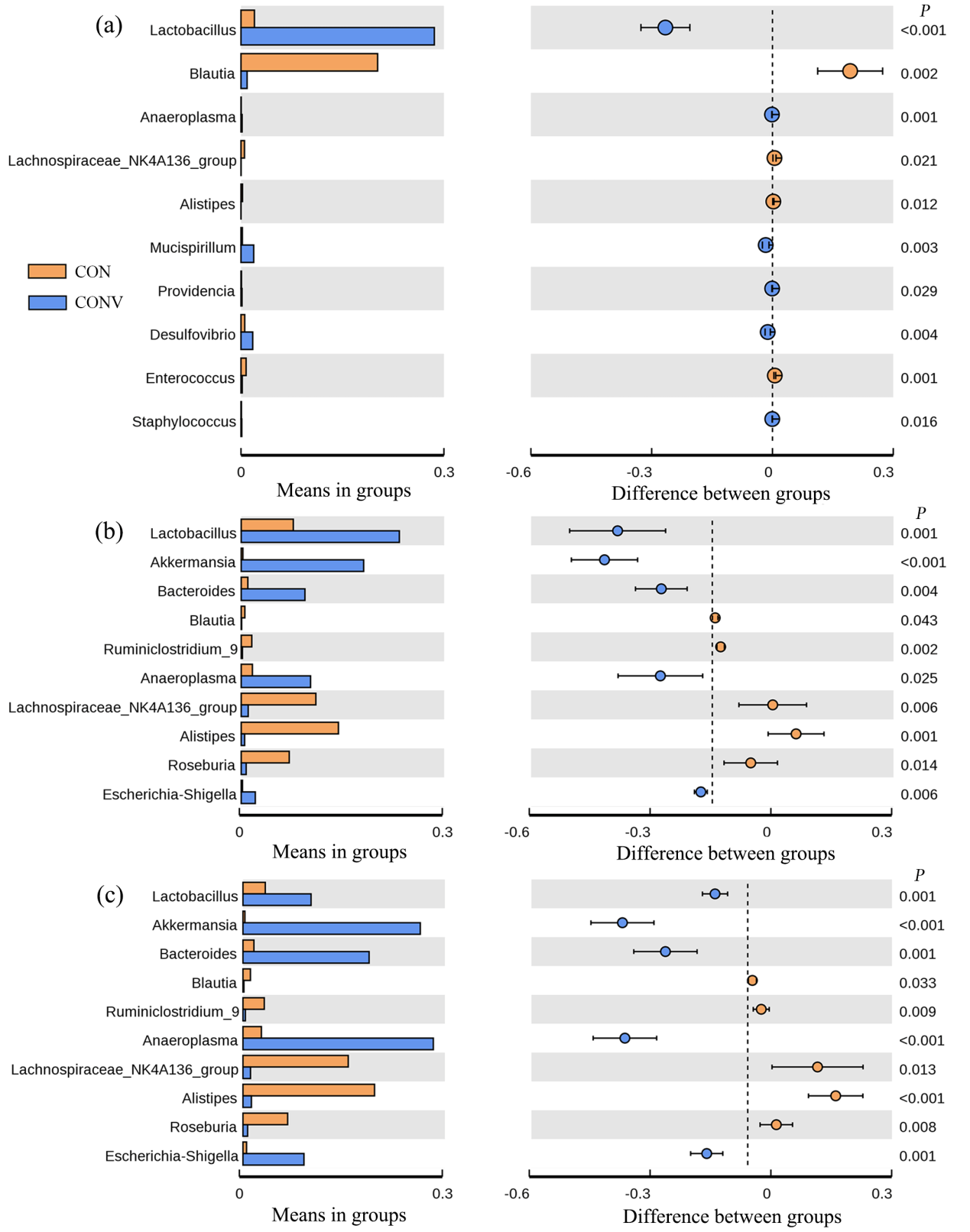


**Figure S7.** Vancomycin exposure alters the gut microbiota composition in normal healthy mice. Top 10 microbes that significantly altered between normal control (CON) and vancomycin-treated CON (CONV) mice at **(a)** 3, **(b)** 7 and **(c)** 11 weeks (*n*=6 mice per group). The difference between two groups was determined by two-tailed unpaired student’s t test with Bonferroni correction.


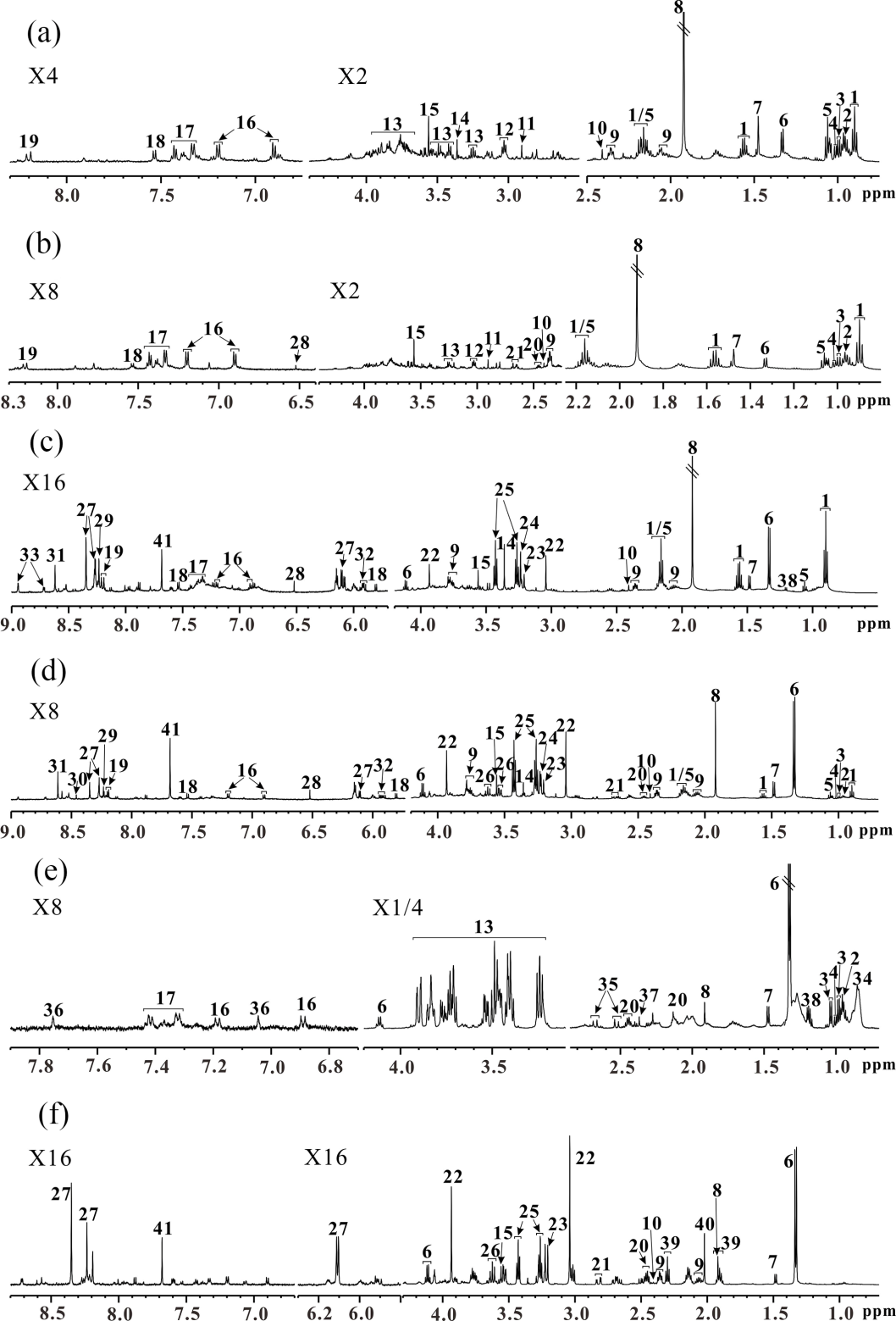


**Figure S8.** NMR-based metabolomic profiling. Typical 600 MHz ^1^H NMR spectra obtained from **(a)** feces, **(b)** caecum contents, **(c)** caecum tissues, **(d)** colonic tissues, **(e)** serum, and **(f)** hippocampus in mice. Metabolite assignment: 1, butyrate; 2, leucine; 3, valine; 4, isoleucine; 5, propionate; 6, lactate; 7, alanine; 8, acetate; 9, glutamate; 10, succinate; 11, trimethylamine; 12, ethylmalonate; 13, glucose; 14, methanol; 15, glycine; 16, tyrosine; 17, phenylalanine; 18, uracil; 19, hypoxanthine; 20, glutamine; 21, aspartate; 22, creatine; 23, choline; 24, phosphocholine; 25, taurine; 26, myo-inositol; 27, inosine; 28, fumarate; 29, adenine; 30, formate; 31, AMP; 32, uridine; 33, nicotinate/niacinamide; 34, LDL/VLDL; 35, citrate; 36, 1-methyhistidine; 37, pyruvate; 38, 3-hydroxybutyrate; 39, γ-aminobutyrate; 40, N-acetylaspartate; 41, unknown.


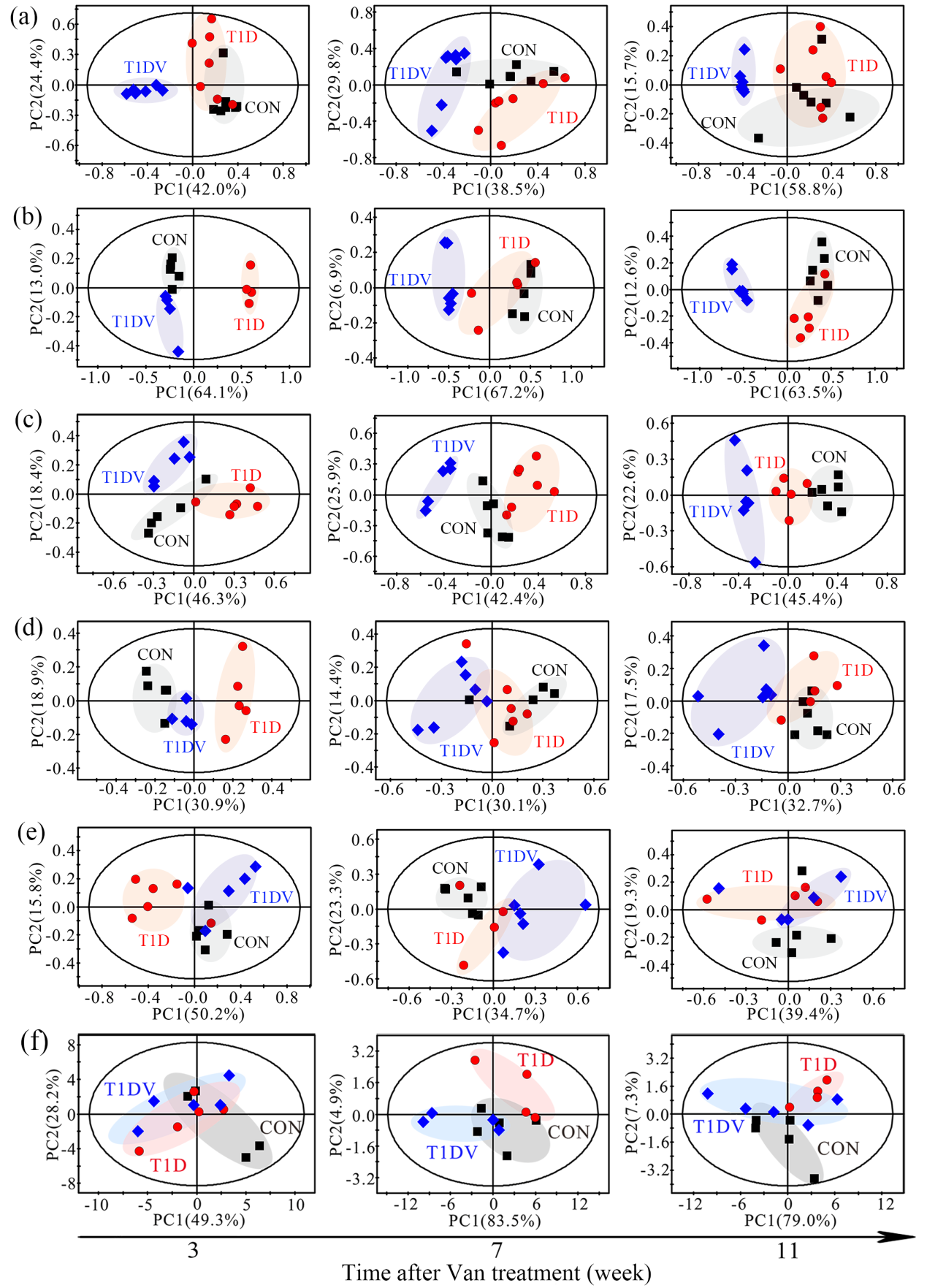


**Figure S9.** Unsupervised metabolic pattern analysis. PCA-based classification using the metabolomic profiling derived from **(a)** feces, **(b)** caecum contents, **(c)** caecum tissues, **(d)** colonic tissues, **(e)** serum, and **(f)** hippocampus in normal control (CON), type 1 diabetic (T1D) and vancomycin-treated T1D (T1DV) mice at 3, 7 and 11 weeks (*n*=4-7 mice per group).


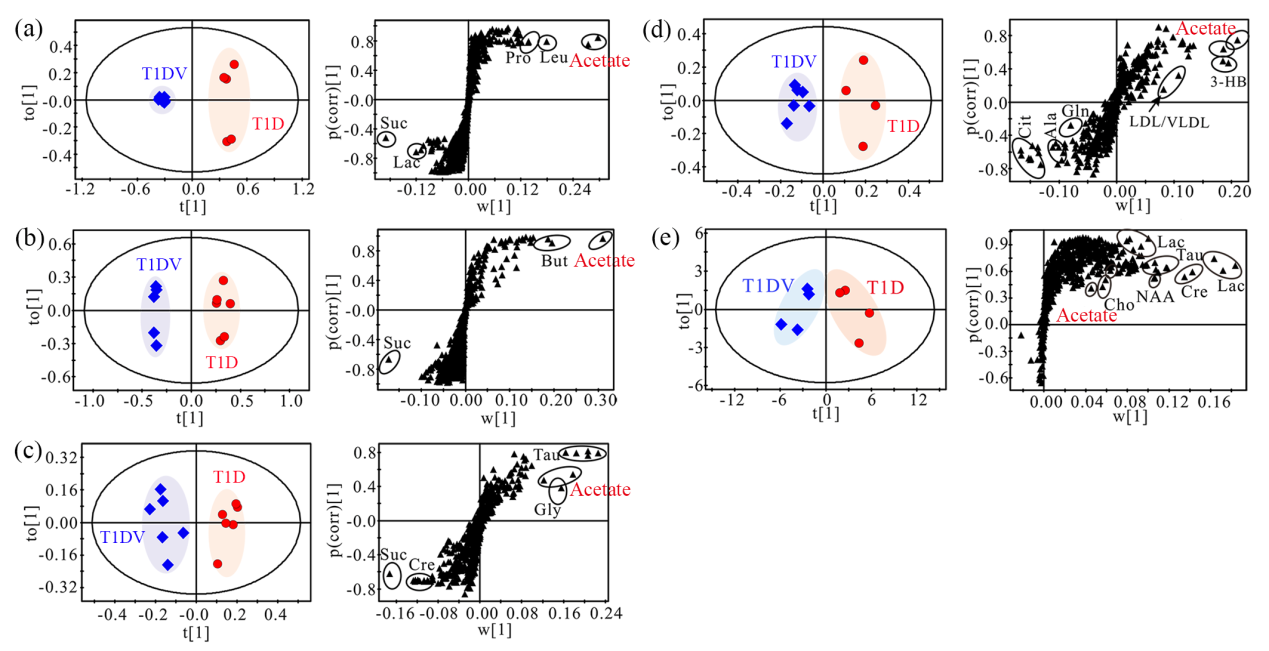


**Figure S10.** Supervised metabolic pattern analysis. OPLS-DA-based classification and the corresponding S-plot using the metabolomic profiling derived from **(a)** caecum contents, **(b)** caecum tissues, **(c)** colonic tissues, **(d)** serum, and **(e)** hippocampus between type 1 diabetic (T1D) and vancomycin-treated T1D (T1DV) mice at 7 weeks (*n*=4-6 mice per group). Metabolite: Ala, alanine; But, butyrate; Cit, citrate; Cre, creatine; Cho, choline; Gly, glycine; Gln, glutamine; Suc, succinate; Tau, taurine; Lac, lactate; Leu, leucine; LDL/VLDL, low-density lipoprotein/very-low-density lipoprotein; NAA, N-acetylaspartate; Pro, propionate; 3-HB, 3-hydroxybutyrate.


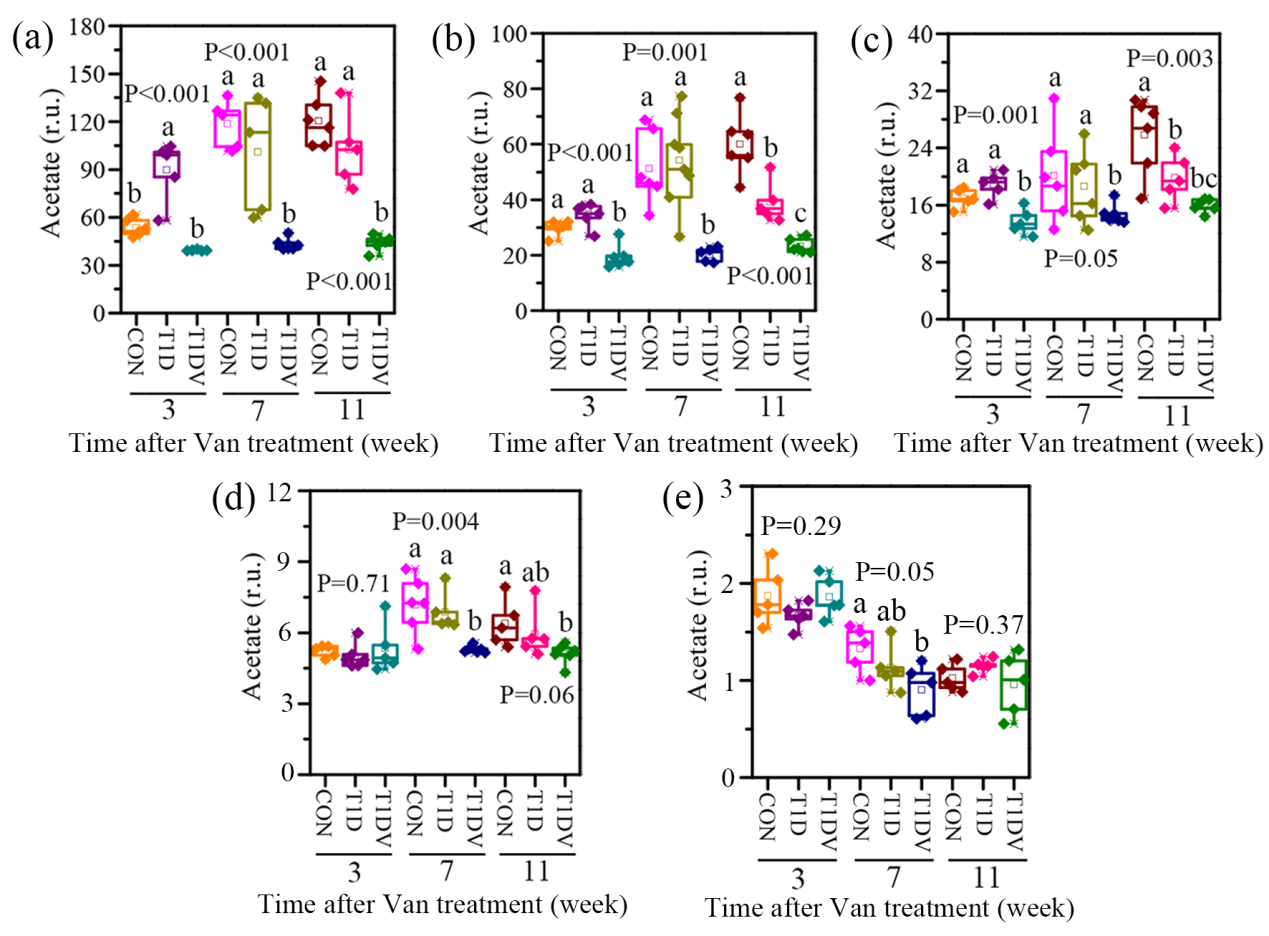


**Figure S11.** Vancomycin exposure decreases the level of acetate in type 1 diabetic (T1D) mice. Changes in the relative concentration of acetate in **(a)** caecum contents, **(b)** caecum tissues, **(c)** colonic tissues, **(d)** serum, and **(e)** hippocampus of normal control (CON), T1D and vancomycin-treated T1D (T1DV) mice at 3, 7 and 11 weeks. Data are presented as mean±s.d. of *n*=6-8 mice per group. The differences among three groups were analyzed by one-way ANOVA with Bonferroni’s multiple comparisons test, and data with different lowercase codes are significantly different (P < 0.05).


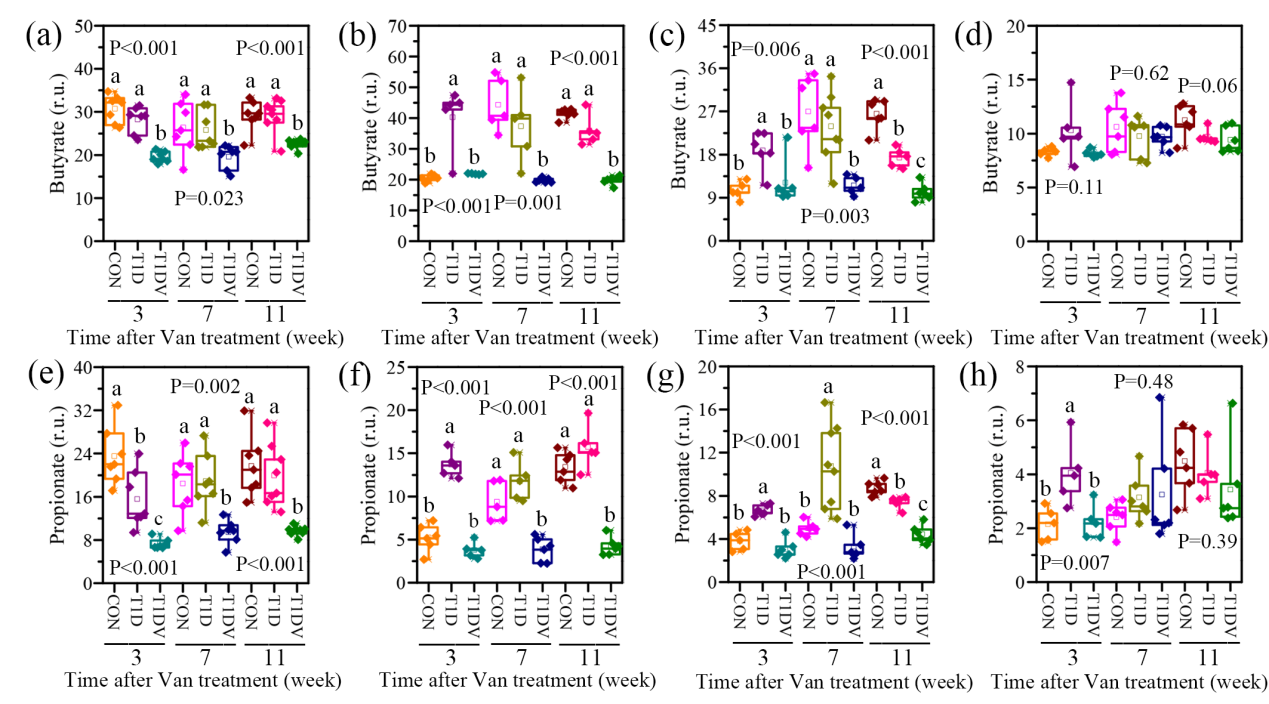


**Figure S12.** Vancomycin exposure decreases the levels of butyrate and propionate in type 1 diabetic (T1D) mice. The changes of butyrate and propionate levels in **(a, e)** feces, **(b, f)** caecum contents, **(c, g)** caecum tissues, and **(d, h)** colonic tissues of normal control (CON), T1D and vancomycin-treated T1D (T1DV) mice at 3, 7 and 11 weeks. Data are shown as mean±s.d. of *n*=6-8 mice per group. The differences among three groups were analyzed by one-way ANOVA with Bonferroni’s multiple comparisons test, and data with different lowercase codes are significantly different (P < 0.05).


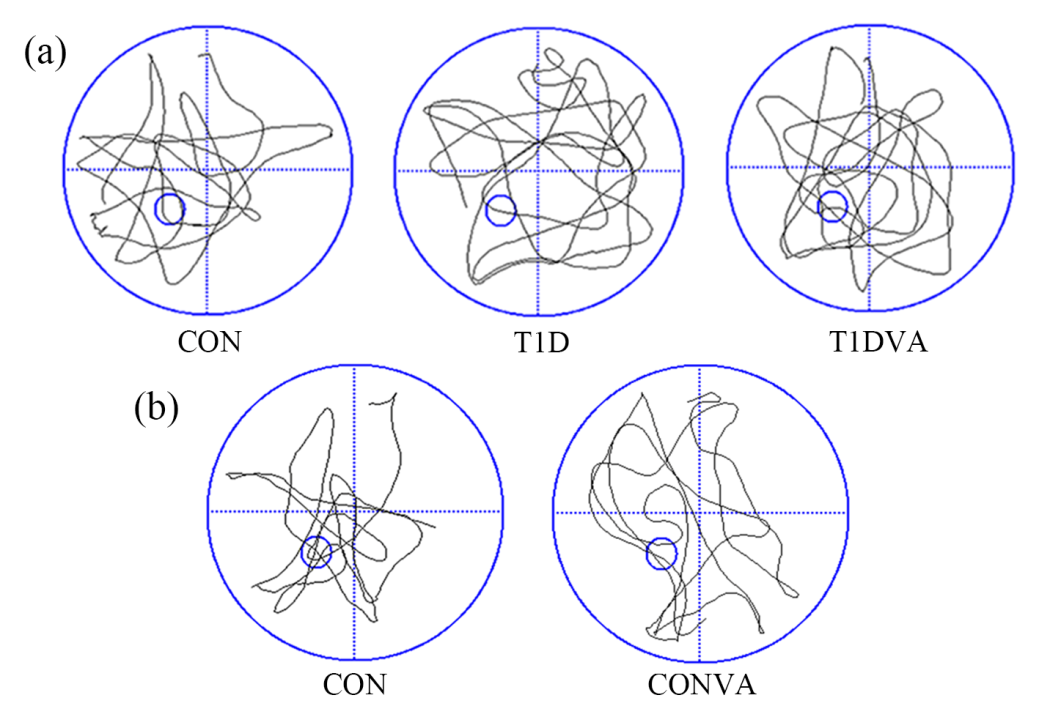


**Figure S13.** The Morris water maze (MWM) test. **(a)** After a 1-week acclimation, mice were injected with streptozocin for 5 consecutive days to develop diabetic 1 diabetic (T1D) mice and then treated with vancomycin (Van) plus acetate (T1DVA). The MWM test was used to evaluate learning and memory ability in T1D mice at 7 weeks after Van treatment. This figure shows swimming path during the test period in normal control (CON), T1D and T1DVA mice at 7 weeks. **(b)** After a 1-week acclimation, normal control (CON) mice were treated with Van plus acetate (CONVA) and then their learning and memory ability was assessed by the MWM test at 11 weeks after Van treatment. This figure shows swimming path during the test period in CON and CONVA mice at 11 weeks.


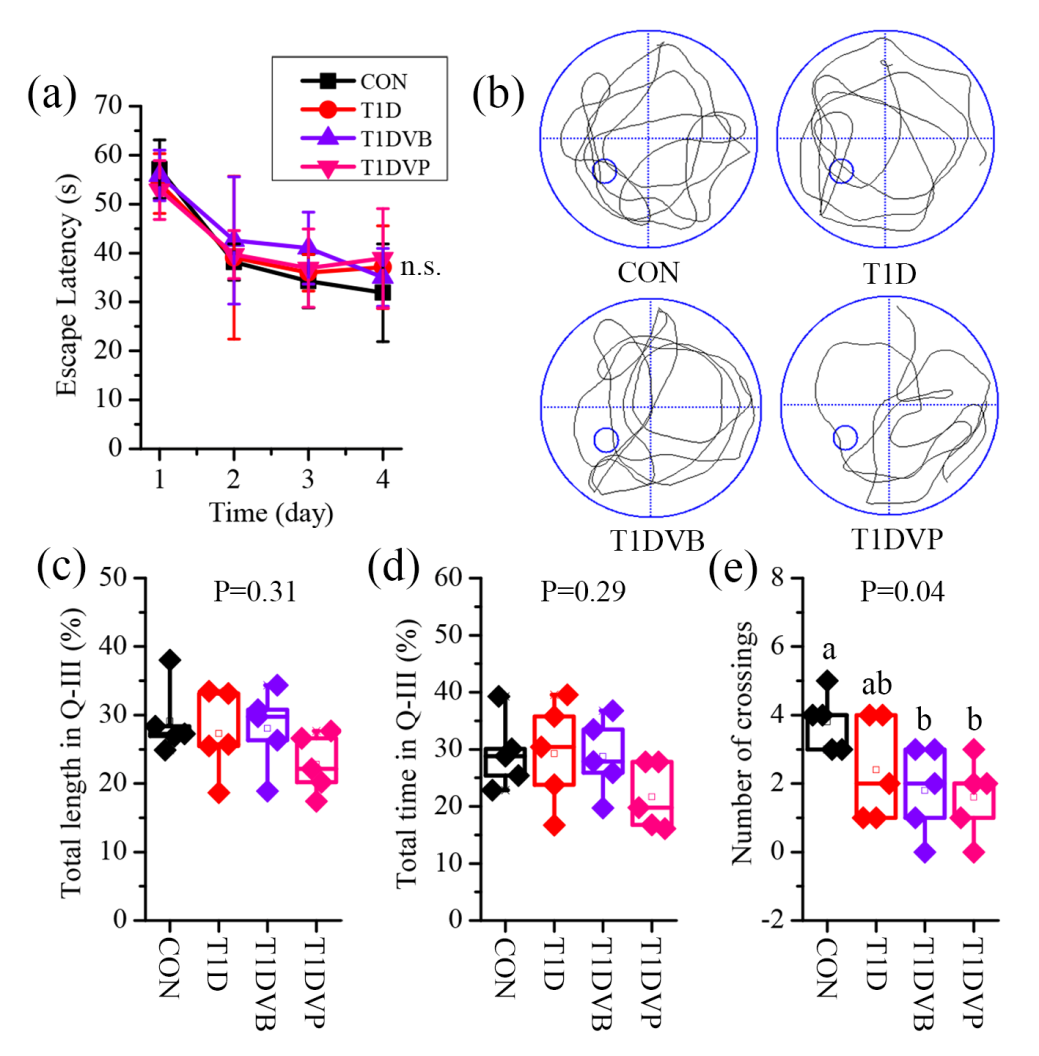


**Figure S14.** The effects of exogenous butyrate and propionate supplements on learning and memory ability in vancomycin-treated T1D (T1DV) mice. **(a)** Escape latency during the training period in normal control (CON), diabetic 1 diabetic (T1D), butyrate-fed T1DV (T1DVB) and propionate-fed T1DV (T1DVP) mice at 7 weeks. **(b)** Swimming path, **(c)** percentage of total swimming length in the Q-III area (original platform quadrant), **(d)** percentage of total swimming time in the Q-III area, and **(e)** the number of crossings over the original platform location during the test period in CON, T1D, T1DVB and T1DVP mice at 7 weeks. Data are shown as mean±s.d. of *n*=5 mice per group. Time-series data were analyzed by repeated measures one-way ANOVA followed by Bonferroni’s multiple comparisons test. The differences among four groups were analyzed by one-way ANOVA with Bonferroni’s multiple comparisons test, and data with different lowercase codes are significantly different (P < 0.05).


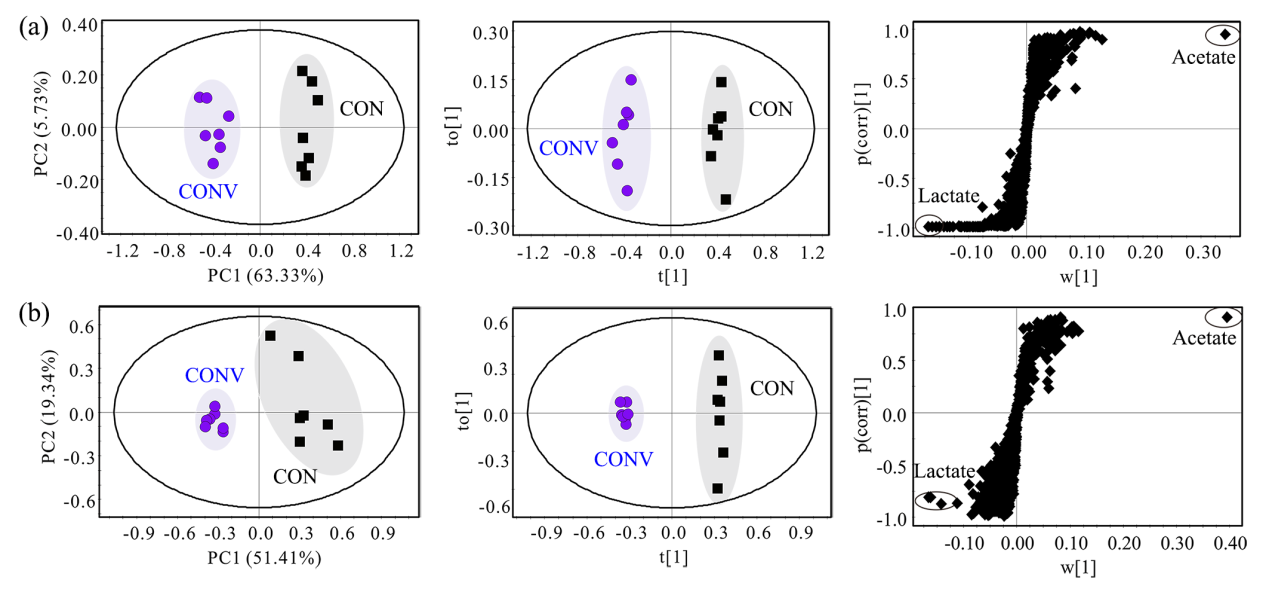


**Figure S15.** Metabolomics data analysis. PCA-and OPLS-DA-based classifications and the corresponding S-plot using faecal metabolomic profiling between normal control (CON) and vancomycin-treated CON (CONV) mice at **(a)** 3 and **(b)** 7 weeks (*n*=7 mice per group).


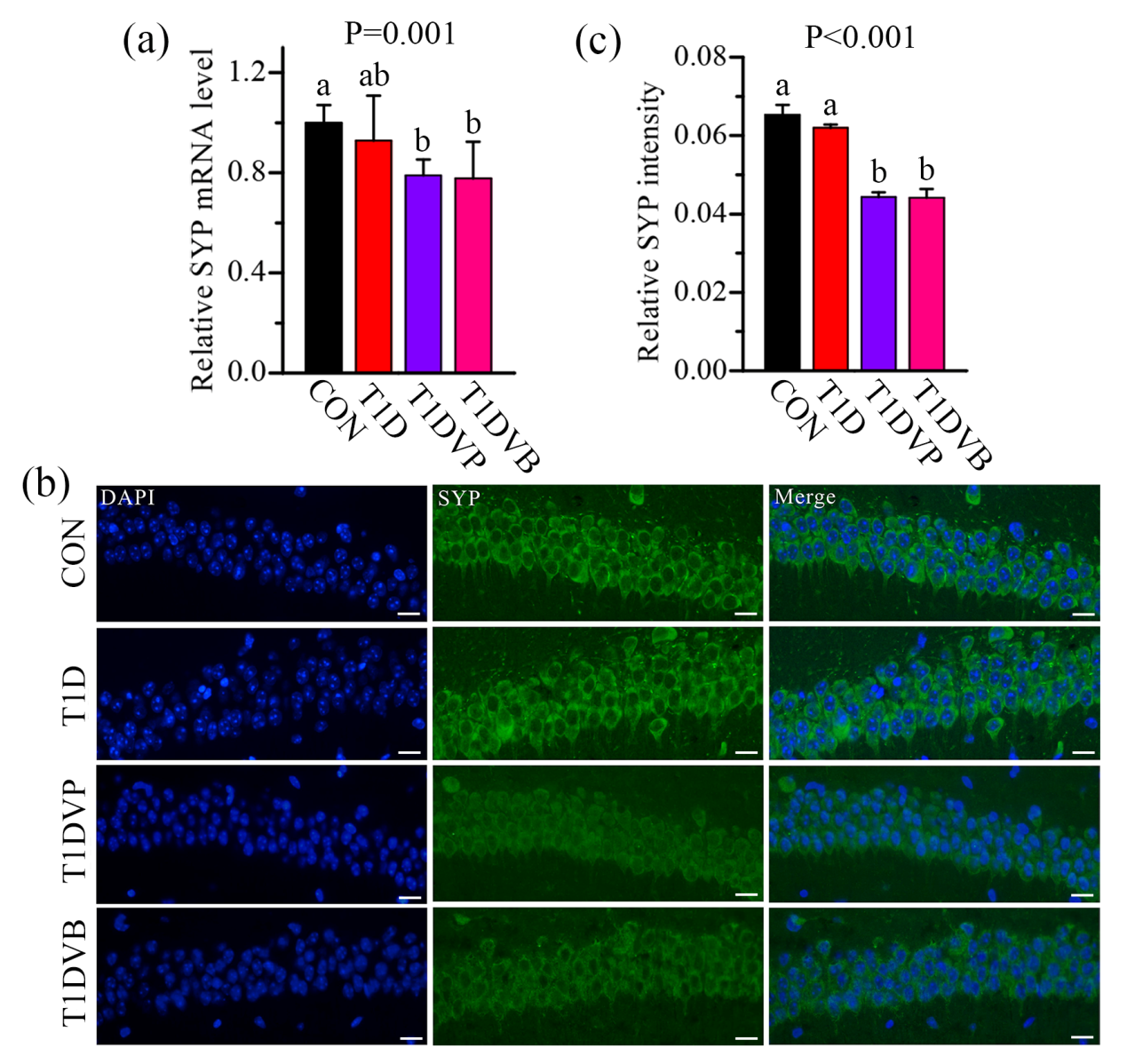


**Figure S16.** The effects of exogenous butyrate and propionate supplements on hippocampal SYP level in vancomycin-treated T1D (T1DV) mice. **(a)** Relative SYP mRNA expression level in hippocampus of normal control (CON), diabetic 1 diabetic (T1D), butyrate-fed T1DV (T1DVB) and propionate-fed T1DV (T1DVP) mice at 7 weeks. **(b, c)** Representative SYP immunostaining and the quantified relative SYP intensity in hippocampal CA1 region of CON, T1D, T1DVP and T1DVB mice. Scale bar = 400 μm. Data are shown as mean±s.d. of *n*=5 mice per group. The differences among four groups were analyzed by one-way ANOVA with Bonferroni’s multiple comparisons test, and data with different lowercase codes are significantly different (P < 0.05).


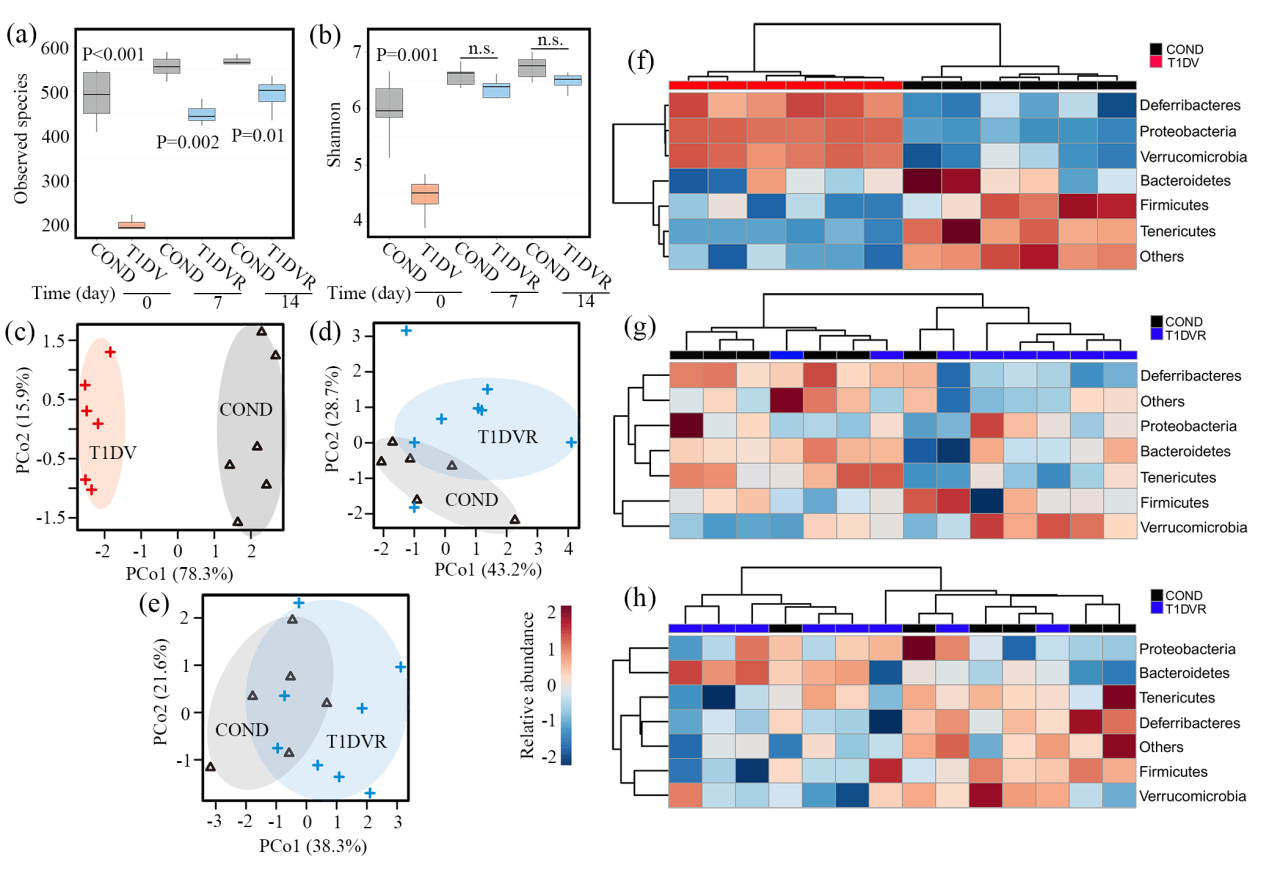


**Figure S17.** Fecal microbiota transplant (FMT) reshapes the gut microbiota in vancomycin-treated type 1 diabetic mice. After a 1-week acclimation, mice were injected with streptozocin for 5 consecutive days to develop type 1 diabetic (T1D) mice and then administered with vancomycin (Van) for 7 weeks. Subsequently, faecal material from healthy age-matched donor mice (COND) was transferred to Van-treated T1D (T1DV) recipients (T1DVR) for 14 consecutive days. Changes in **(a)** the observed species and **(b)** Shannon index in T1DV mice during FMT. PCoA-based classification using the gut microbiome in T1DV mice after FMT at **(c)** 0, **(d)** 7 and **(e)** 14 days. Relative abundance of the gut microbiota at the phylum level in feces of T1DV mice after FMT at **(f)** 0, **(g)** 7 and **(h)** 14 days. Cluster analysis was conducted by using Ward’s method and Euclidean distance. Data are shown as mean±s.d. of *n*=6-8 mice per group. The difference between two groups was determined by two-tailed unpaired student’s t test with Bonferroni correction. n.s., no significant difference.
